# An exceptionally preserved Late Devonian actinopterygian provides a new model for primitive cranial anatomy in ray-finned fishes

**DOI:** 10.1098/rspb.2015.1485

**Published:** 2015-10-07

**Authors:** Sam Giles, Laurent Darras, Gaël Clément, Alain Blieck, Matt Friedman

**Affiliations:** 1 Department of Earth Sciences, University of Oxford, South Parks Road, Oxford OX1 3AN, UK; 2 313 Rue Nationale, Appt. 96, Nœux-les-Mines 62290, France; 3 Centre de Recherches sur la Paléobiodiversité et les Paléoenvironnements (CR2P, UMR 7207), Sorbonne Universités, MNHN, CNRS, UPMC-Paris 6, Muséum National d'Histoire Naturelle, CP 38, 57 Rue Cuvier, 75231 Paris cedex 05, France; 4 Université de Lille—Sciences et technologies, UFR Sciences de la Terre, UMR 8198 ‘EvoEcoPaleo’ du CNRS, Villeneuve d'Ascq cedex 59655, France

**Keywords:** Actinopterygii, braincase, computed tomography, gill skeleton, Osteichthyes, Frasnian

## Abstract

Actinopterygians (ray-finned fishes) are the most diverse living osteichthyan (bony vertebrate) group, with a rich fossil record. However, details of their earliest history during the middle Palaeozoic (Devonian) ‘Age of Fishes' remains sketchy. This stems from an uneven understanding of anatomy in early actinopterygians, with a few well-known species dominating perceptions of primitive conditions. Here we present an exceptionally preserved ray-finned fish from the Late Devonian (Middle Frasnian, *ca* 373 Ma) of Pas-de-Calais, northern France. This new genus is represented by a single, three-dimensionally preserved skull. CT scanning reveals the presence of an almost complete braincase along with near-fully articulated mandibular, hyoid and gill arches. The neurocranium differs from the coeval *Mimipiscis* in displaying a short aortic canal with a distinct posterior notch, long grooves for the lateral dorsal aortae, large vestibular fontanelles and a broad postorbital process. Identification of similar but previously unrecognized features in other Devonian actinopterygians suggests that aspects of braincase anatomy in *Mimipiscis* are apomorphic, questioning its ubiquity as stand-in for generalized actinopterygian conditions. However, the gill skeleton of the new form broadly corresponds to that of *Mimipiscis*, and adds to an emerging picture of primitive branchial architecture in crown gnathostomes. The new genus is recovered in a polytomy with Mimiidae and a subset of Devonian and stratigraphically younger actinopterygians, with no support found for a monophyletic grouping of *Moythomasia* with Mimiidae.

## 1. Introduction

Actinopterygians (ray-finned fishes) and sarcopterygians (lobe-finned fishes inclusive of tetrapods) together comprise Osteichthyes (bony vertebrates), representing more than 99% of living vertebrate species richness [1]. Over 50% of this diversity—some 32 000 species [1]—is contained within Actinopterygii. Despite modern prominence, details of early actinopterygian history remain obscure. Fewer than 20 species are described from the roughly 55 Myr of the Devonian (in comparison to more than 200 sarcopterygians), and no definitive examples are known from the Silurian despite their predicted occurrence [2]. Detailed understanding of the structure of early actinopterygians, particularly that of the character-rich endoskeleton, has changed little since the exhaustive description and accompanying discussion of acid-prepared material of *Mimipiscis* and *Moythomasia* from the Frasnian Gogo Formation of Australia over three decades ago [3,4]. Actinopterygians discovered or redescribed subsequent to this monographic effort have been represented almost exclusively by the external skeleton, and surprisingly little progress has been made in our understanding of the relationships of Devonian forms [5–11], or indeed in the broader problem of joining Palaeozoic lineages to the stems of modern actinopterygian radiations [12]. Meanwhile, new insights have revised past perceptions about the relationships and evolution of sarcopterygians and gnathostomes more generally [2,13–20], with similar advances occurring in our understanding of extant actinopterygians (e.g. the rediscovery of Holostei [21] and increased resolution of the percomorph ‘bush’[22]). Indeed, *Mimipiscis* remains the only Devonian actinopterygian for which both the internal and external structure has been exhaustively described and illustrated. Unsurprisingly, it is the default exemplar of primitive ray-fin anatomy (e.g. [23–26]), the most common comparator when referring to early actinopterygians or osteichthyans (e.g. [16,19,20,27,28]), and a template for anatomical restoration of other early actinopterygians (e.g. [29]). Furthermore, although the account provided by Gardiner [3] provides a wealth of morphological information, disarticulation of the specimens during preparation led to a loss of important spatial information relating to complex structures like the gill skeleton.

Here we present an exceptionally preserved ray-finned fish skull from the Late Devonian (Middle Frasnian, *ca* 373 Ma) Ferques Formation of northern France. Known from a single articulated specimen, this material comprises a near-complete dermal cranium, braincase and mandibular, hyoid and gill arches. With the exception of the heavily compressed *Cheirolepis* [30], this taxon presents the only Devonian actinopterygian neurocranium reported outside of Australia. Our investigation of this specimen using micro-computed tomography (µCT) helps to illustrate key aspects of anatomy lost in acid-prepared material of Gogo actinopterygians, most notably the three-dimensional geometry and articulation of the skull and branchial arches.

## 2. Material and methods

### (a) Micro-computed tomography scanning

MGL 1245 (electronic supplementary material, figure S1) was scanned at the AST-RX platform of the Muséum national d'Histoire naturelle, Paris, France, using a GE Sensing & Inspection Technologies Phoenix x-ray CT scanner v-tome-x L 240–180 (http://www.ums2700.mnhn.fr/ast-rx/ressources) at 80 kV and 500 µA (18.69 µm voxel size). Data were segmented using Mimics v. 15.01 (biomedical.materialise.com/mimics; Materialise, Leuven, Belgium). Meshes were exported as .ply surface files to Blender (blender.org) for image and video acquisition [31].

### (b) Phylogenetic dataset assembly and analyses

Our dataset derives from Choo [4], with substantial additions of new taxa (25) and characters (106) and corrections reflecting new anatomical details (electronic supplementary material). Inclusion of non-osteichthyan outgroups permits investigation of the branching pattern of actinopterygians and sarcopterygians, as well as osteichthyans more broadly.

An equally weighted parsimony analysis was conducted using a heuristic search in PAUP* v. 4.0b10 [32] (1000 random addition sequences, five trees held at each step, maxtrees set to automatically increase, nchuck = 10 000, chuckscore = 1, the tree bisection and reconstruction strategy enabled). Six characters were ordered. Taxonomic equivalence [33] was assessed using Claddis [34], and all taxa were found to comprise unique character combinations. The outgroup was constrained using the topology [*Entelognathus* [*Acanthodes, Cladodoides, Ozarcus*][ingroup]]. Bootstrap values were calculated in PAUP (1000 replicates of a heuristic search, tree branching and reconstruction strategy enabled, 25 replicates, five trees held at each step, rearrlimit = 50 000 000, limitperrep = yes, nchuck = 10 000, chuckscore = 1). Bremer support values were calculated using PRAP2 [35].

## 3. Systematic palaeontology

Osteichthyes Huxley, 1880 [36].

Actinopterygii Cope, 1887 [37].

*Raynerius splendens* n. gen. et sp.

### (a) Etymology

Generic name after Dorothy Rayner (1912–2003) for her contributions to palaeoichthyology, particularly those relating to actinopterygian neurocrania (e.g. [38,39]). Specific name reflects the exceptional preservation and articulated nature of the specimen.

### (b) Material

MGL 1245, Natural History Museum of Lille, Nord, France, representing a nearly complete skull (electronic supplementary material, figure S1). Collected by Mr Christian Loones (amateur palaeontologist, member of the Société Géologique du Nord) in April 2000.

### (c) Locality and horizon

Up per part of the Grey Member, Ferques Formation, La Parisienne quarry (now flooded), Pas-de-Calais, France. Specimen from lowermost *Ag. triangularis* Conodont Zone [40, fig. 2; p. 15], equivalent to late *Palmatolepis hassi* Zone (*ca* 373 Ma, Frasnian) [41].

### (d) Diagnosis

Actinopterygian characterized by the following combination of characters: ornamentation on skull roof formed of short, serrated ridges; porous ornamentation on lower jaw, gular and clavicle; dermosphenotic lacking anterior ramus; median gular equal in length to lateral gulars; distinct jugal notch; braincase lacking a spiracular canal; no ascending processes on parasphenoid.

## 4. Description

### (a) Skull

**Figure 1.**
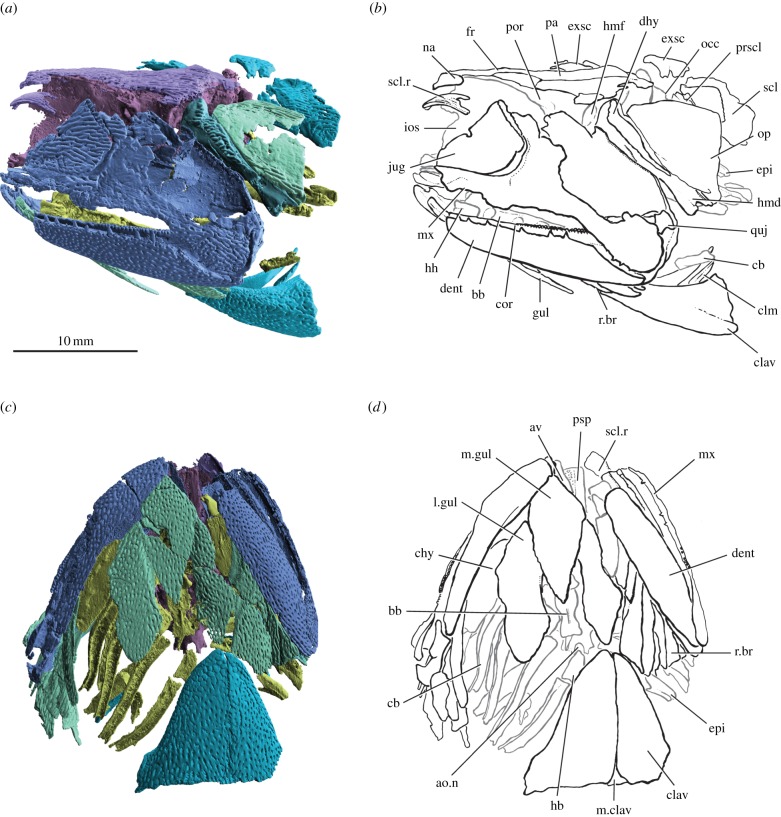
*Raynerius splendens* n. gen. et sp. (*a*) Rendering and (*b*) interpretive drawing of specimen in left lateral view. (*c*) Rendering and (*d*) interpretive drawing of specimen in ventral view; skeletal elements interior to the dermal bones (i.e. braincase, gill skeleton) shown in grey. Abbreviations: a.on, aortic notch; av, accessory vomer; bb, basibranchial; cb, ceratobranchial; chy, ceratohyal; clav, clavicle; clm, cleithrum; cor, coronoid; dent, dentary; dhy, dermohyal; epi, epibranchial; exsc, extrascapular; fr, frontal; gul, gular; hb, hypobranchial; hh, hypohyal; hmd, hyomandibula; hmf, hyomandibular facet; ios, interorbital septum; jug, jugal; l.gul, lateral gular; m.clav, median clavicle; m.gul, median gular; mx, maxilla; na, nasal; op, operculum; pa, parietal; por, postorbital process; prscl, presupracleithrum; psp, parasphenoid; quj, quadratojugal; r.br, branchiostegal ray; scl, supracleithrum; scl.r, sclerotic ring. Colour coding of the skeleton: blue, cheek and jaw; purple, skull roof and sclerotic ossicle; pink, braincase; dark green, hyomandibula; light green, operculogular system; turquoise, shoulder girdle; yellow, gill skeleton.

**Figure 2.**
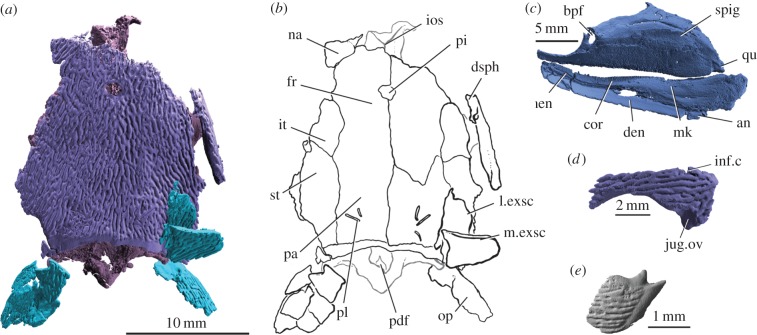
*Raynerius splendens* n. gen. et sp*.* (*a*) Rendering and (*b*) interpretive drawing of skull roof. (*c*) Right palatoquadrate and lower jaw in medial view, showing basipterygoid fenestra and ossified mentomeckelian cartilage. (*d*) Right dermosphenotic in lateral view, showing well-developed posterior limb and lack of anterior limb. (*e*) Scale in external view, showing ganoin ridges, anterodorsal process and dorsal peg. Abbreviations: an, angular; bpf, basipterygoid fenestra; cor, coronoid; den, dentary; dsph, dermosphenotic; fr, frontal; inf.c, infraorbital canal; ios, interorbital septum; it, intertemporal; jug.ov, overlap area for jugal; l.exsc, lateral extrascapular; m.exsc, median extrascapular; men, mentomeckelian ossification; mk, Meckel's cartilage; na, nasal; op, operculum; pa, parietal; pdf, posterior dorsal fontanelle; pi, pineal foramen; pl, pit line; qu, quadrate; spig, spiracular groove; st, supratemporal. For a key to colours see figure 1.

The skull roof comprises paired frontals, parietals, intertemporals and supratemporals (conventional actinopterygian terminology is applied here [5,7]). Short, vermiform ridges covering the skull roof (figures 1*a* and 2*a*) resemble those of *Moythomasia durgaringa* [3]. Anterior, middle and posterior pit lines are borne on the parietals, and the pineal foramen is open. Two pairs of extrascapulars rest on the posterior margin of the parietals (figure 2*a,b*). Unlike *Cheirolepis* [42], *Osorioichthys* [43], *Tegeolepis* [44] and *M. durgaringa* [3], the lateral extrascapular extends beyond the posterior flange of the supratemporal to contribute to the lateral margin of the skull roof (electronic supplementary material, figure S10). The dermosphenotic possesses an elongate posterior limb, typical for early actinopterygians [29], but unusually lacks an anterior limb (figure 2*d*). The deep, crescentic jugal bears a blunt dorsal edge (figure 1*a,b*). A notch marks the orbital margin of the jugal, as in *Cheirolepis* [42]. The quadratojugal is present as a separate ossification, but it is unclear whether a quadratojugal pit line is present.

The cleaver-shaped maxilla broadly resembles that of other early actinopterygians and primitive sarcopterygians [14,45]. The palatoquadrate contacts a pronounced shelf on the medial surface of the maxilla, and comprises a rectangular postorbital plate and a short and relatively deep suborbital limb (figure 2*c*). A large basipterygoid fenestra pierces the anterior part of the postorbital plate. Dentigerous dermal bone covers three quarters of the medial face of the palatoquadrate (including the suborbital limb), but separate ossifications cannot be identified. As in *Mimipiscis*, the dorsal margin of the dermopalatine is straight, not undulating as in *Gogosardina* [10]. A denticulated, blade-shaped accessory vomer lies adjacent to the suborbital limb of the palatoquadrate.

**Figure 3.**
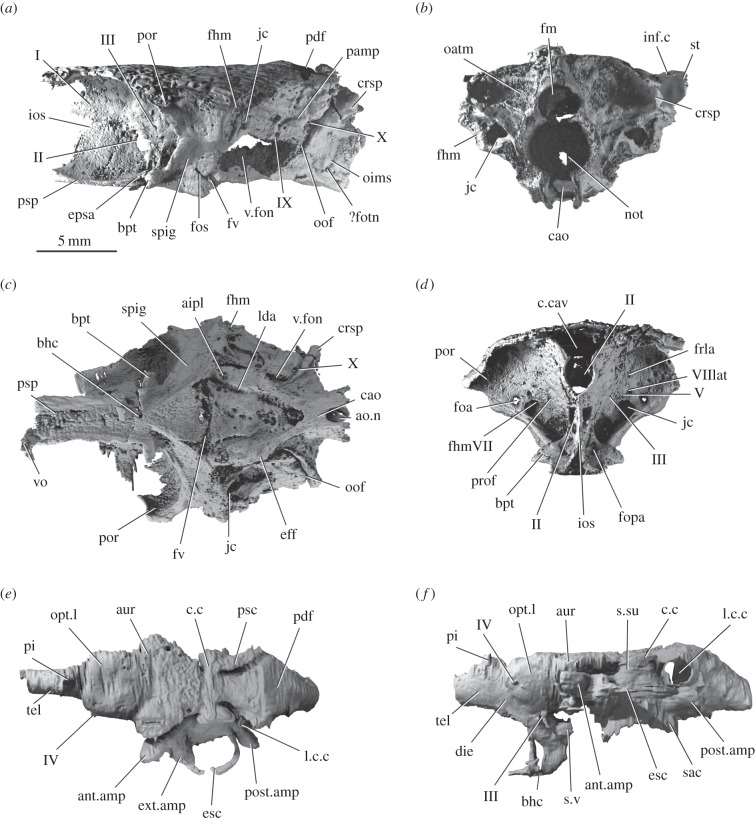
Braincase and endocast of *Raynerius splendens* n. gen. et sp. (*a*) Braincase in left lateral, (*b*) posterior, (*c*) ventral and (*d*) anterior view. (*e*) Partial endocast in dorsal and (*f*) lateral view; some details of labyrinth and saccular chamber unresolvable due to poor mineralization. Abbreviations: aip1, articulation of the first infrapharyngobranchial; ant.amp; ampulla of the anterior semicircular canal; ao.n, aortic notch; aur, cerebellar auricle; bhc, buccohypophysial canal; bpt, basipterygoid process; cao, canal for the dorsal aorta; c.cav, cranial cavity; c.c, crus commune; crsp, craniospinal process; die, diencephalon; eff, efferent arteries; epsa, efferent pseudobranchial; esc, external semicircular canal; ext.amp, ampulla of the external semicircular canal; fhm, hyomandibular facet; fhmVII, hyomandibular branch of the facial nerve; fm, foramen magnum; foa, foramen for orbital artery; fopa, ophthalmic artery; fos, otico-sphenoid fossa; ?fotn, foramen for otic nerve; frla, ramus lateralis accessiorus; fv; vental fissure; inf.c, infraorbital canal; ios, interorbital septum; jc, jugular canal; l.c.c, lateral cranial canal; lda, lateral dorsal aorta; not, notochordal tunnel; oatm, origin of anterior trunk muscles; oims, origin of intermuscular septum; oof, oticoccipital fissure; opt.l, optic lobe; pamp, parampullary process; pdf, posterior dorsal fontanelle; pi, pineal foramen; por, postorbital process; post.amp, ampulla of the posterior semicircular canal; prof, profundus nerve; psc, posterior semicircular canal; psp, parasphenoid; sac, sacculus; spig, spiracular groove; s.su, sinus superior; st, supratemporal; s.v, saccus vasculosus; tel, telencephalon; v.fon, vestibular fontanelle; vo. vomer; I, olfactory nerve; II, optic nerve; III, oculomotor nerve; IV, trochlear nerve; V, trigeminal nerve; VIIlat, lateralis trunk of facial nerve; IX, glossopharyngeal nerve; X, vagus nerve.

The long, narrow parasphenoid is denticulated along most of its length (figure 3*c*), although the lateral extent of this denticulated portion on the basicranium is unclear. The anterior margin is incompletely preserved, but does not appear tripartite. *Raynerius* lacks ascending processes, like *Cheirolepis* [30,42] and *Mimipiscis* [3,4]. Parotic toothplates are not preserved, and are presumed absent.

The stout dentary does not taper significantly rostrally, unlike *Mimipiscis* and *Gogosardina* [3,4,10]. Acrodin caps are present on the largest teeth, and a surangular is present (electronic supplementary material, figure S6). The Meckelian cartilage is ossified as a single element. The mandibular canal traces the ventral margin of the jaw along its entire length, and reaches the anterior margin of the mandible (electronic supplementary material, figures S3 and S6). The ornament on the dentary is smooth and punctuated by regular large pores, similar to that of *M. durgaringa* and *M. nitida* [3,46] (figure 1*a*, electronic supplementary material, figure S6).

The large clavicles comprise a gently convex ventral plate and slender dorsal blade (figure 1). Ornament on the dorsal blade is tuberculate, and in some places almost oak-leaf shaped. Elsewhere on the clavicles the ornament is of the porous type seen on the lower jaw. A small portion of the interclavicle is preserved, resembling that of *M. durgaringa* [3] in shape and ornament.

### (b) Braincase and endocast

The braincase is undistorted and almost completely mineralized, although the bone is thin in areas of the orbital wall and occiput. It differs from that of *Mimipiscis*, the only other contemporaneous taxon for which the braincase is well known, in several features. The very short aortic canal bifurcates at the level of the vagus nerve, and its posterior margin is notched (figure 3*c*). This resembles the incompletely described condition in *M. durgaringa* [3,47, fig. 5b] and *Gogosardina* [10, fig. 9a], but contrasts sharply with the extensive aortic canal of *Mimipiscis* [3, fig. 50]. Grooves for the lateral dorsal aortae in *Raynerius* are elongate, and a shallow, laterally directed groove marks the path of an epibranchial artery (figure 3*c*). The otic and occipital regions account for a larger proportion of the braincase than in *Mimipiscis*. The otico-occipital fissure is open along its length, and terminates ventrally in a large vestibular fontanelle and dorsally in a small, ovoid posterior dorsal fontanelle (figures 2*a* and 3*a*). The vestibular fontanelles are not continuous with the ventral fissure. The dorsolateral process of the occiput is formed by a robust craniospinal process. The dorsal roof of the braincase is smooth; unlike in Carboniferous [39] and younger taxa [48], no fossa bridgei is present (electronic supplementary material, figure S5).

The postorbital process is broad, but far less laterally produced than that in *Mimipiscis* (figure 3*a,c,d*). The hyomandibular facet sits lateral to and above the jugular groove on the posterior face of the postorbital process. The wide spiracular groove shows no hint of the enclosing canal seen in *Moythomasia* ([3,46], M Coates 2014, personal communication) and stratigraphically younger forms [49]. A break in the lateral commissure suggests the presence of an otico-sphenoid fissure (figure 3*a*), previously identified only in the Gogo ray fins [3,4,10]. The two walls of the narrow interorbital septum join at the midline (figure 3*d*). The optic nerves enter the orbit through a single confluent opening. Weak mineralization of the posteroventral corner of the orbit makes the extent of the posterior myodome uncertain, but a distinct depression is clearly present. The pituitary vein canal is somewhat enlarged, more similar to the condition in *M. durgaringa* than *Mimipiscis* [3].

Only a partial endocast can be described due to weak mineralization. Olfactory tracts and bulbs are not preserved. The hypophysial chamber projects posteriorly, and is continuous ventrally with the buccohypophyseal canal (figure 3*f*, electronic supplementary material, figure S5). Although narrower than the cerebellum, the optic lobes are somewhat wider than in *Mimipiscis* (figure 3*e*), but do not approach the proportions seen in Carboniferous and statigraphically younger taxa [6,48–50]. Cerebellar auricles are pronounced, although it is unclear whether a corpus was developed. The semicircular canals are only partially preserved, but the crus commune of the anterior and posterior canals clearly extends dorsal to the endocranial roof (figure 3*f*), matching the general osteichthyan condition [50]. The large lateral cranial canal projects through the loop of the posterior canal (figure 3*f*, electronic supplementary material, figure S5). As in *Mimipiscis*, the ampulla of this canal is not confluent with the cranial cavity.

### (c) Hyoid and branchial skeleton

**Figure 4.**
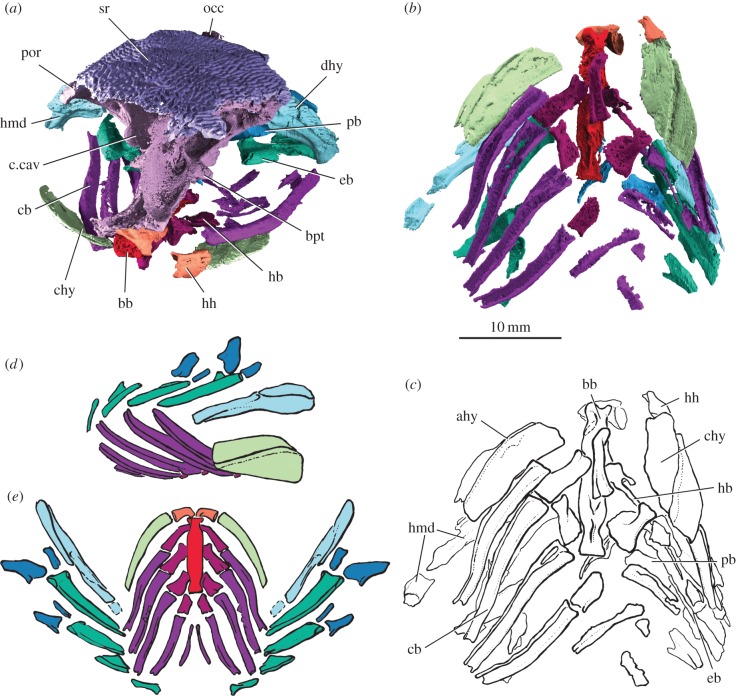
Branchial and hyoid skeleton of *Raynerius splendens* n. gen. et sp. (*a*) Braincase and skull roof in anterolateral view showing articulation of gill skeleton. (*b*) Rendering and (*c*) interpretive drawing of branchial and hyoid skeleton in ventral view. (*d*) Reconstruction of the branchial and hyoid skeleton in right lateral and dorsal (*e*) view, with the presence of an interhyal (dotted) inferred (hyomandibula moved out of life position in lateral reconstruction and inferred interhyal not shown so as not to obscure the branchial arches). Abbreviations: ahy, afferent hyoid artery; bb, basibranchial; bpt, basipterygoid process; c.cav, cranial cavity; cb, ceratobranchial; chy, ceratohyal; dhy, dermohyal; eb, epibranchial; hb, hypobranchial; hh, hypohyal; hmd, hyomandibula; occ, occiput; pb, pharyngobranchial; por, postorbital process; sr, skull roof. For a key to colours see figure 1.

The slender hyomandibula is fused to the dermohyal, and is pierced by a single canal for the hyomandibular nerve (figures 1*a* and 4, electronic supplementary material, figure S3). As in other early actinopterygians including *Cheirolepis*, *Mimipiscis* and *Moythomasia*, there is no opercular process. The distal part of the hyomandibula is missing completely on the left side of the specimen due to breakage, but the relevant region of its antimere is present. Breakage precludes identification of an interhyal. The ceratohyal is roughly rectangular, with a groove for the afferent hyoid artery on its lateral face (figure 4). As the ceratohyal is unmineralized distally it is unclear whether one or two ossifications were present, although the general shape recalls that of Triassic taxa such as *Pteronisculus* ([48], fig. 43), rather than the more slender ossification of *Mimipiscis* ([3], fig. 106). The distal end of the hypohyal, which would have articulated with the ceratohyal, is inflated to approximately twice the size of the proximal end. As in *Mimipiscis* [3], the basibranchial is a single midline ossification, with clear articular areas for the hypobranchials (figure 4*b,c*). Unlike in *Mimipiscis*, the first hypobranchial does not appear to have shared an articular facet with the hypohyal, instead meeting a long, slender facet on the lateral face of the basibranchial. Five ceratobranchials are present. Ceratobranchials 1–4 are long and slender, and grooved along their ventral surfaces. Ceratobranchial 5 is also grooved, but is much shorter and narrower (figure 4*b,c*). The first four ceratobranchials articulate ventrally with hypobranchials and dorsally with epibranchials. The epibranchials are typically shorter than the ceratobranchials, and each bears a dorsal groove (figure 4*e*). The first two branchial arches include an anteriorly directed infrapharyngobranchial and anterodorsally directed suprapharyngobranchial, which articulate ventrally with facets on the epibranchials (figure 4*d,e*). Narrow, elongate toothplates, each bearing a large number of minute denticles, are present on the ceratobranchials and epibranchials.

### (d) Scales

Scales possess a well-developed peg, socket and anterodorsal process (figure 2*e*). Ornament consists of anterodorsally oriented ridges that do not anastomose. These ridges bear fine, accessory striations (electronic supplementary material, figure S8), as in *M. durgaringa*, *M. nitida* [51], and *Mimipiscis toombsi* [4]. Lateral line scales bear a distinct pore (electronic supplementary material, figure S7).

## 5. Phylogenetic results

**Figure 5.**
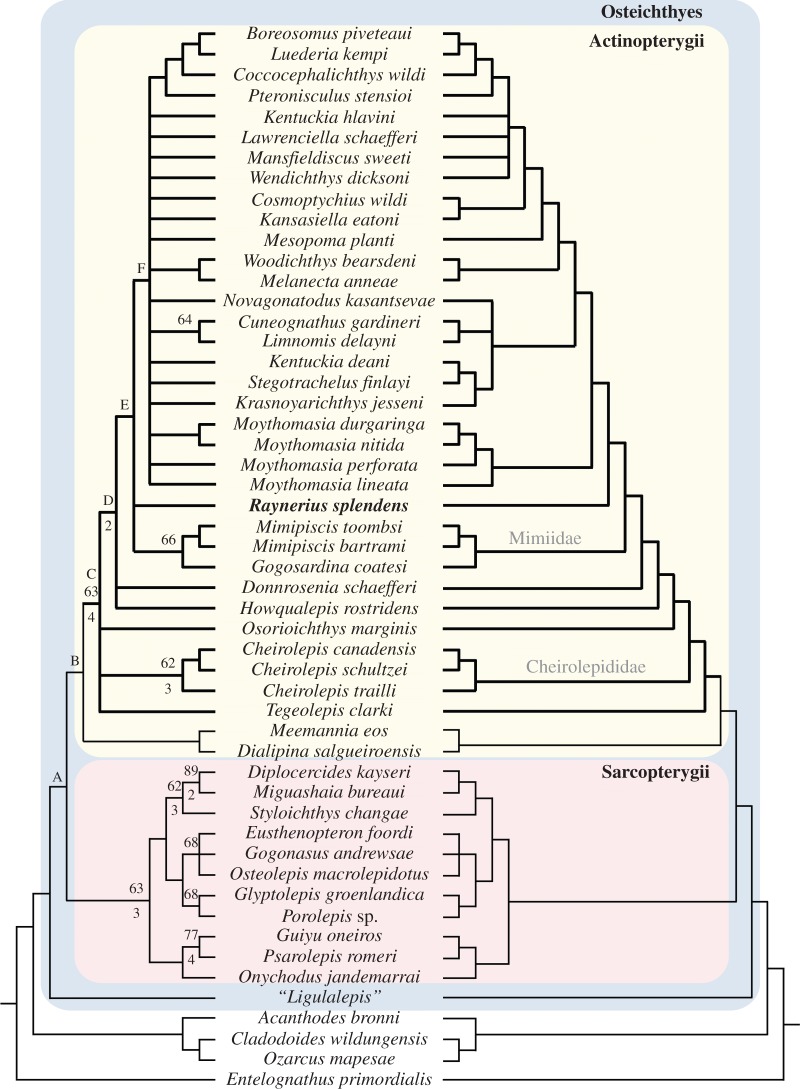
Phylogenetic placement of *Raynerius splendens* n. gen. et sp. Strict consensus of 384 most parsimonious trees. Numbers above nodes represent bootstrap support; numbers below indicate Bremer support values. Branches leading to unequivocal actinopterygians (i.e. those agreed by previous analyses to be ray-finned fishes) are in bold. Apomorphic features at selected nodes are as follows (numbers refer to character list in electronic supplementary material, Supplementary Notes; full optimization tree provided in the electronic supplementary material): A, c.90 eyestalk absent; c.126 elongate olfactory tracts. B, c.31 extrascapular does not reach lateral edge of skull roof; c.174 pelvic fin has long-based insertion. C, c.25 dermosphenotic with posterior ramus; c.55 median gular present; c.58 expanded dorsal lamina of maxilla; c.77 two infradentaries; c. 179 ventral scutes between hypochordal lobe of caudal fin and anal fin; c.181 one dorsal fin. D, c. 12 posterior nostril in complete communication with orbit; c.31 extrascapular reaches lateral edge of skull roof; c.162 presupracleithrum present. E, c.112 dorsal aortic canal notched posteriorly; c.174 pelvic fin insertion short based; c.175 epichordal lobe of caudal fin absent; c.182 anal fin shifted posteriorly relative to dorsal. F, c. 18 frontal longer than parietal; c. 53 dorsal-most branchiostegal deeper than adjacent ray; c. 98 spiracle housed in canal; c. 120 ascending processes of the parasphenoid present.

The equally weighted analysis yielded 384 most parsimonious trees (MPTs) of 520 steps (consistency index (CI) = 0.373; retention index (RI) = 0.673; rescaled consistency index (RCI) = 0.251). The strict consensus is poorly resolved, and both bootstrap percentages (BP) and Bremer decay indices (BDI) are generally low for clades within Actinopterygii; character transformations for key nodes are given in figure 5. The neurocranium attributed to *Ligulalepis* is resolved as a stem osteichthyan, a placement commonly recovered in other analyses [16,18–20,25,52,53]. *Dialipina* and *Meemannia* are resolved as stem actinopterygians, but support is weak. Although *Dialipina* was originally described as an actinopterygian [54] and placed as such in some cladistic analyses [2,55,56], more recent studies have favoured interpretation as a stem osteichthyan [16,18–20,25,52,53]. *Meemannia* has variably been resolved as a stem sarcopterygian [25,56,57] or, more recently, as a stem osteichthyan [19,53].

The clade uniting all unequivocal actinopterygians is better supported (Clade C; BP = 63%; BDI = 4). *Cheirolepis*, *Osorioichthys* and *Tegeolepis* form an unresolved basal polytomy with a clade including all remaining unequivocal actinopterygians (Clade D). The earliest diverging lineages of this extended radiation are *Howqualepis* and *Donnrosenia*. The third branch subtended by this basal polytomy contains *Raynerius*, which lies in polytomy with the Mimiidae *sensu* Choo [4] and a clade uniting all remaining Devonian taxa and stratigraphically younger forms (Clade E). This radiation includes an unresolved clade (Clade F) containing many Late Devonian and all post-Devonian taxa sampled in the analysis.

Reweighting by RCIs yielded 63 trees of length 134.15 (CI = 0.370; RI = 0.668; RCI = 0.247). A strict consensus agrees with much of the basic structure of the equally weighted solution, but offers increased resolution. Most significant are the placement of *Tegeolepis* as the sister taxon of all unamibiguous actinopterygians; resolution of *Donnrosenia*, *Howqualepis* and *Osorioichthys* as successively more distant outgroups to Clade E of the unweighted solution; the monophyly of the species of *Moythomasia* to the clear exclusion of *Raynerius*; and the implication that no fewer than three lineages of ray-finned fishes survived the Hangenberg Event to contribute to their meteoric rise in the Carboniferous [12,26,58,59].

## 6. Discussion

### (a) Early actinopterygian interrelationships: instability and stability

Failure to resolve consistent clades across different analyses reflects a major obstacle to a more complete understanding of the earliest stages of ray-finned fish evolution. Sub-clades have been recognized in individual analyses, but are generally poorly supported and rarely re-appear in subsequent studies despite broadly overlapping taxon and/or character sets. This chronic inconsistency leads us to suggest that the low resolution of our equally weighted tree represents a realistic summary of current understanding of the relationships of the earliest actinopterygians. We fail to find support for many putative clades of Devonian actinopterygians, including: *Howqualepis* + *Mimipiscis* [5]; *Tegeolepis* + *Howqualepis* [8]; *Howqualepis* + *Donnrosenia* [9,10]; Mimiidae + *Moythomasia* [4]. This is not limited to suprageneric cohorts, with our analysis also calling into question the monophyly of individual genera, including *Kentuckia* (cf. [8,50]) and *Moythomasia* (*contra* [51]).

However, several aspects of our solution are in broad agreement with those in other studies, suggesting a stable phylogenetic core providing a foundation for subsequent work. These include: the unambiguous monophyly of *Cheirolepis* and its basal position along with taxa such as *Osorioichthys* and *Tegeolepis*; placement of *Howqualepis* and *Donnrosenia* between these early diverging lineages and ‘stegotrachelid’-grade forms; a monophyletic Mimiidae comprising *Mimia* and *Gogosardina*; a sister-group relationship between *Limnomis* and *Cuneognathus*, and between *M. durgaringa* and *M. nitida*.

The lack of phylogenetic resolution for early actinopterygians contrasts strikingly with the situation for sarcopterygians, where some clades have been recognized for over a century (e.g. [60,61]). This discrepancy likely reflects both biological and historical factors. It is clear that early actinopterygians were ecologically conservative relative to sarcopterygians, where the profound functional specializations of groups like lungfishes and coelacanths make their isolation as clades relatively straightforward. Compounding this is the rarity and small size of many early actinopterygians in comparison to their lobe-finned contemporaries [26,59]. Both factors impede the study of endoskeletal anatomy that is such an important source of systematic characters, although µCT is beginning to address this deficiency [30,50]. Taxonomic practice might exacerbate the difference in the apparent maturity of phylogenetic solutions for these two clades, manifest in generic undersplitting in actinopterygians relative to sarcopterygians. For example, material assigned to *Cheirolepis* ranges in age from Eifelian to Frasnian, with individual species showing conspicuous differences in fin insertion, and the pattern, proportion and ornament of dermal bones, comparable to that separating closely related lobe-fin genera (e.g. *Tristichopterus*, *Eusthenopteron*, *Jarvikina*), inclusion of these different species in a single generic lineage exaggerates the apparent absence of higher-level clades among Devonian actinopterygians. However, attribution of new species to less secure genera—and the corresponding illusion of monophyly in the absence of analysis—also represents a challenge for early actinopterygian systematics. *Moythomasia* is such a case, exacerbated by limited material of both the type species [51] and many attributed ones [62].

### (b) Primitive actinopterygian endocranial structure reconsidered

*Raynerius* provides a rare window on details of internal cranial anatomy in early actinopterygians, with important bearing on general conditions for this clade and, when considered alongside more complete data for early sarcopterygians, osteichthyans more generally. *Raynerius* makes the greatest impact with respect to the braincase and gill skeleton, structures best known in *Mimipiscis* among Devonian actinopterygians. More limited data are available for a few additional genera, where structure is often interpreted in the light of *Mimipiscis* [29]. *Raynerius* represents an alternative model for interpreting these taxa, and a test of the generality of conditions in *Mimipiscis*.

Differences between *Mimipiscis* and *Raynerius* in the character-rich gill skeleton are relatively slight. This, along with close apparent agreement between structure in *Mimipiscis* and *Moythomasia*, suggests a relatively conserved branchial architecture geometry in early actinopterygians. This broad anatomical congruence helps to corroborate the model of crown gnathostome gill skeleton geometry laid out by Pradel *et al.* [63]: arches arranged as a chevron; paired hypohyals connect to the ceratohyals; hypobranchials oriented anteriorly; two anteriorly directed pharyngobranchials (supra- and infrapharyngobranchials) present in at least the first two arches; at least four of the arches articulate ventrally with the basibranchial.

In contrast to this conserved picture of gill-arch structure, *Raynerius* highlights neurocranial disparity among the earliest ray-finned fishes. The short and wide aortic canal of *Raynerius*, also present in *M. durgarina* ([47], fig. 5b) and *Gogosardina* ([10], fig. 9a), seems to reflect the primitive condition for canal-bearing actinopterygians; the dorsal aorta is unenclosed in the most primitive actinopterygians (e.g. *Cheirolepis* and *Howqualepis*) [30] and sarcopterygians [64,65]. By contrast, the elongate, narrow aortic canal of *Mimipiscis* ([3], fig. 50) appears apomorphic, as do its short lateral dorsal aortae, small vestibular fontanelles and narrow lateral commissure [3]. The orbital region in *Mimipiscis* is very long, representing over half of neurocranial length. Furthermore, the postorbital process extends laterally and somewhat anteriorly to partially enclose the back of the orbit, a feature not seen in *Raynerius* or other early actinopterygians. In sum, emerging data from *Raynerius* and other early actinopterygians [30] paint a picture of autapomorphic neurocranial anatomy in *Mimipiscis*. The revelation of underappreciated braincase variation in Devonian ray-fins highlights this structure as a new source of characters for teasing out interrelationships, and contradicts previous views that braincases were too conservative in nature for systematic use ([5], p. 137). Future descriptions of braincase and endocast anatomy of *Gogosardina* [10] and *M. durgaringa* [3], as well as understudied Carboniferous actinopterygians, will help to further clarify relationships among the Palaeozoic antecedents of a diverse modern vertebrate group.

## 7. Summary

We describe an exceptionally preserved early actinopterygian, *Raynerius splendens* n. gen. et sp., from the Frasnian (approx. 373 Ma) of France, the only early representative of the clade where character-rich anatomical systems like the neurocranium, mandibular arch and branchial arches are preserved in three-dimensions with positional information largely intact. *Raynerius* complements the best-known Devonian actinopterygian, *Mimipiscis* [3,4], helping to provide a more representative picture of primitive conditions of the ray-fin braincase and gill skeleton. Despite this, relationships among early ray-finned fishes remain largely unresolved. However, there are broad areas of stability, with most uncertainty relating to poorly known or highly incomplete taxa, or species with largely non-overlapping character sets (i.e. dermal versus endoskeletal). µCT study of this unique but fractured specimen of *Raynerius* highlights recovery of detailed information from incomplete material, but the technique has also yielded important new information for complete, seemingly well-known taxa [30,50]. Applied more widely, this approach might provide the critical raw data needed to better constrain early branching events in the actinopterygian tree of life [26].

## Supplementary Material

Supplementary Notes

## Supplementary Material

Supplementary Figures

## Supplementary Material

Data Matrix

## Data accessibility

The datasets supporting this article are available in Dryad (doi:10.5061/dryad.n66h4).

## References

[1] Eschmeyer WN (ed.). 2015 Catalog of fishes: genera, species, references. See http://researcharchive.calacademy.org/research/ichthyology/catalog/fishcatmain.asp (accessed 26/05/2015).

[2] Zhu M, Zhao W, Jia L, Lu J, Qiao T, Qu Q 2009 The oldest articulated osteichthyan reveals mosaic gnathostome characters. Nature 458, 469–474. (10.1038/nature07855) 19325627

[3] Gardiner BG 1984 The relationships of the palaeoniscid fishes, a review based on new specimens of *Mimia* and *Moythomasia* from the Upper Devonian of Western Australia. Bull. Br. Mus. Nat. Hist. 37, 173–428.

[4] Choo B 2011 Revision of the actinopterygian genus *Mimipiscis* (=*Mimia*) from the Upper Devonian Gogo formation of Western Australia and the interrelationships of the early Actinopterygii. Earth Environ. Sci. Trans. R. Soc. Edinb. 102, 77–104. (10.1017/S1755691011011029)

[5] Gardiner BG, Schaeffer B 1989 Interrelationships of lower actinopterygian fishes. Zool. J. Linn. Soc. 97, 135–187. (10.1111/j.1096-3642.1989.tb00550.x)

[6] Coates MI 1999 Endocranial preservation of a Carboniferous actinopterygian from Lancashire, UK, and the interrelationships of primitive actinopterygians. Phil. Trans. R. Soc. Lond. B 354, 435–462. (10.1098/rstb.1999.0396)

[7] Gardiner BG, Schaeffer B, Masserie JA 2005 A review of the lower actinopterygian phylogeny. Zool. J. Linn. Soc. 144, 511–525. (10.1111/j.1096-3642.2005.00181.x)

[8] Friedman M, Blom H 2006 A new actinopterygian from the Famennian of East Greenland and the interrelationships of Devonian ray-finned fishes. J. Paleontol. 80, 1186–1204. (10.1666/0022-3360%282006%2980%5B1186%3AANAFTF%5D2.0.CO%3B2)

[9] Long JA, Choo B, Young GC 2008 A new basal actinopterygian from the Middle Devonian Aztec Siltstone of Antarctica. Antarct. Sci. 20, 393–412. (10.1017/S0954102008001144)

[10] Choo B, Long JA, Trinajstic K 2009 A new genus and species of basal actinopterygian fish from the Upper Devonian Gogo Formation of Western Australia. Acta Zool.-Stockholm *Suppl* 90, 194–210. (10.1111/j.1463-6395.2008.00370.x)

[11] Swartz BA 2009 Devonian actinopterygian phylogeny and evolution based on a redescription of *Stegotrachelus finlayi*. Zool. J. Linn. Soc. 156, 750–784. (10.1111/j.1096-3642.2009.00505.x)

[12] Sallan LC 2014 Major issues in the origins of ray-finned fish (Actinopterygii) biodiversity. Biol. Rev. 89, 950–971. (10.1111/brv.12086) 24612207

[13] Cloutier R, Ahlberg PE 1996 Morphology, characters, and the interrelationships of basal sarcopterygians. In Interrelationships of fishes (eds Stiassny MLJ, Parenti LR, Johnson GD), pp. 445–479. New York, NY: Academic Press.

[14] Zhu M, Yu X, Janvier P 1999 A primitive fossil fish sheds light on the origin of bony fishes. Nature 397, 607–610. (10.1038/17594)

[15] Maisey JG 2001 A primitive chondrichthyan braincase from the Middle Devonian of Bolivia. In Major events in early vertebrate evolution: palaeontology, phylogeny, genetics and development (ed. Ahlberg PE), pp. 263–288. London, UK: Taylor & Francis.

[16] Brazeau MD 2009 The braincase and jaws of a Devonian ‘acanthodian’ and modern gnathostome origins. Nature 457, 305–308. (10.1038/nature07436) 19148098

[17] Pradel A, Maisey JG, Tafforeau P, Janvier P 2009 An enigmatic gnathostome vertebrate skull from the Middle Devonian of Bolivia. Acta Zool. 90, 123–133. (10.1111/j.1463-6395.2008.00350.x)

[18] Davis SP, Finarelli JA, Coates MI 2012 *Acanthodes* and shark-like conditions in the last common ancestor of modern gnathostomes. Nature 486, 247–250. (10.1038/nature11080) 22699617

[19] Zhu M et al. 2013 A Silurian placoderm with osteichthyan-like marginal jaw bones. Nature 502, 188–193. (10.1038/nature12617) 24067611

[20] Giles S, Friedman M, Brazeau MD 2015 Osteichthyan-like cranial conditions in an Early Devonian stem gnathostome. Nature 520, 82–85. (10.1038/nature14065) 25581798 PMC5536226

[21] Grande L 2010 An empirical synthetic pattern study of gars (Lepisosteiformes) and closely related species, based mostly on skeletal anatomy. The resurrection of Holostei. Am. Soc. Ichthyol. Herpetol. Spec. Publ. 6, 1–871.

[22] Near TJ et al. 2013 Phylogeny and tempo of diversification in the superradiation of spiny-rayed fishes. Proc. Natl Acad. Sci. USA 110, 12 738–12 743. (10.1073/pnas.1304661110) PMC3732986 23858462

[23] Janvier P 1996 Early vertebrates. Oxford, UK: Clarendon Press.

[24] Basden AM, Young GC 2001 A primitive actinopterygian neurocranium from the Early Devonian of southeastern Australia. J. Vertebr. Paleontol. 21, 754–766. (10.1671/0272-4634%282001%29021%5B0754%3AAPANFT%5D2.0.CO%3B2)

[25] Friedman M 2007 *Styloichthys* as the oldest coelacanth: implications for early osteichthyan interrelationships. J. Syst. Palaeontol. 5, 289–343. (10.1017/S1477201907002052)

[26] Friedman M 2015 The early evolution of ray-finned fishes. Palaeontology 58, 213–228. (10.1111/pala.12150)

[27] Forey PL 1998 History of the coelacanth fishes. London, UK: Chapman and Hall.

[28] Zhu M, Yu X 2009 Stem sarcopterygians have primitive polybasal fin articulation. Biol. Lett. 5, 372–375. (10.1098/rsbl.2008.0784) 19324642 PMC2679918

[29] Long JA 1988 New palaeoniscoid fishes from the Late Devonian and Early Carboniferous of Victoria. Mem. Assoc. Australas. Palaeontol. 7, 1–68.

[30] Giles S, Coates MI, Garwood RJ, Brazeau MD, Atwood R, Johanson Z, Friedman M 2015 Endoskeletal structure in *Cheirolepis* (Osteichthyes, Actinopterygii), an early ray-finned fish. Palaeontology. 58, 849–870. (10.1111/pala.12182) 27478252 PMC4950109

[31] Garwood R, Dunlop J 2014 The walking dead: blender as a tool for paleontologists with a case study on extinct arachnids. J. Paleontol. 88, 735–746. (10.1666/13-088)

[32] Swofford DL 2002 PAUP*. Phylogenetic analysis using parsimony (*and other methods). Version 4. Sunderland, MA: Sinauer Associates.

[33] Wilkinson M 1995 Coping with missing entries in phylogenetic inference using parsimony. Syst. Biol. 44, 501–514. (10.2307/2413657)

[34] Lloyd GT 2015 Claddis: an R package for performing disparity and rate analysis on cladistic-type data sets. Online at GitHub. See https://github.com/graemetlloyd/Claddis.

[35] Müller K 2004 PRAP—computation of Bremer support for large data sets. Mol. Phylogenet. Evol. 31, 780–782. (10.1016/j.ympev.2003.12.006) 15062810

[36] Huxley TH 1880 On the applications of the laws of evolution to the arrangement of the Vertebrata and more particularly of the Mammalia. Proc. Zool. Soc. Lond. 43, 649–662.

[37] Cope ED 1887 Geology and palaeontology. Am. Nat. 1887, 1014–1019.

[38] Rayner DH 1948 The structure of certain Jurassic holostean fishes with special reference to their neurocrania. Phil. Trans. R. Soc. Lond. B 233, 287–345. (10.1098/rstb.1948.0006)

[39] Rayner DH 1951 On the cranial structure of an early palaeoniscid, *Kentuckia* gen. nov. Trans. R. Soc. Edinb. 62, 58–83. (10.1017/S0080456800009248)

[40] Brice D 1988 *Le Dévonien de Ferques. Bas-Boulonnais (N.France). Paléontologie - Sédimentologie - Stratigraphie - Tectonique.* Coll. Biostratigraphie du Paléozoïque, Université de Bretagne Occidentale, Brest.

[41] Gradstein FM, Ogg JG, Schmitz M (eds). 2012 The geologic time scale 2012, 2-volume set, 1176 Amsterdam, UK: Elsevier.

[42] Pearson DM, Westoll TS 1979 The Devonian actinopterygian *Cheirolepis* Agassiz. Trans. R. Soc. Edinb. 70, 337–399. (10.1017/S0080456800012850)

[43] Taverne L 1997 *Osorioichthys marginis*, ‘Paléonisciforme’ du Famennien de Belgique, et la phylogénie de Actinoptérygiens dévoniens (Pisces). Bull. Inst. R. Sci. Nat. Belg. Biol. 67, 57–78.

[44] Dunkle DH, Schaeffer B 1973 *Tegeolepis clarki* (Newberry), a palaeonisciform from the Upper Devonian Ohio Shale. Palaeontogr. Abt. A 143, 151–158.

[45] Andrews SM, Long JA, Ahlberg PE, Barwick R, Campbell K 2006 The structure of the sarcopterygian *Onychodus jandemarrai* n. sp. from Gogo, Western Australia: with a functional interpretation of the skeleton. Trans. R. Soc. Edinb. 96, 197–307. (10.1017/s0263593300001309)

[46] Jessen H 1968 *Moythomasia nitida* Gross und *M*. cf. *striata* Gross, Devonische palaeonisciden aus dem oberen Plattenkalk der Bergish-Gladbach-Paffrather Mulde (Rheinisches Schiefergebirge). Palaeontogr. Abt. A 128, 87–114.

[47] Long JA, Trinajstic K 2010 The Late Devonian Gogo Formation Lägerstatte of Western Australia: exceptional early vertebrate preservation and diversity. Ann. Rev. Earth Planet. Sci. 38, 255–279. (10.1146/annurev-earth-040809-152416)

[48] Nielsen E 1942 Studies on Triassic fishes from East Greenland I. *Glaucolepis* and *Boreosomus*. Meddel. Grønl. 146, 1–309.

[49] Poplin C 1974 Étude de quelques Paléoniscidés pennsylvaniens du Kansas. Paris, France: Cahiers de Paléontologie (Section Vertébrés).

[50] Giles S, Friedman M 2014 Virtual reconstruction of endocast anatomy in early ray-finned fishes (Osteichthyes, Actinopterygii). J. Paleontol. 88, 636–651. (10.1666/13-094)

[51] Choo B 2015 A new species of the Devonian actinopterygian *Moythomasia* from Bergisch Gladbach, Germany, and fresh observation on *M. durgaringa* from the Gogo Formation of Western Australia. J. Vertebr. Paleontol. 35, e952817 (10.1080/02724634.2015.952817)

[52] Friedman M, Brazeau MD 2010 A reappraisal of the origin and basal radiation of the Osteichthyes. J. Vertebr. Paleontol. 30, 36–56. (10.1080/02724630903409071)

[53] Dupret V, Sanchez S, Goujet D, Tafforeau P, Ahlberg PE 2014 A primitive placoderm sheds light on the origin of the jawed vertebrate face. Nature 507, 500–503. (10.1038/nature12980) 24522530

[54] Schultze H-P 1968 Palaeoniscoidea-Schuppen aus dem Unterdevon Australiens und Kanadas und aus dem Mitteldevon Spitzbergens. Bull. Br. Mus. Nat. Hist. 16, 343–376.

[55] Schultze H-P, Cumbaa SL 2001 *Dialipina* and the characters of basal osteichthyans. In Major events in early vertebrate evolution (ed. Ahlberg PE), pp. 315–332. London, UK: Taylor & Francis.

[56] Zhu M, Yu X, Wang W, Zhao W, Jia L 2006 A primitive fish provides key characters bearing on deep osteichthyan phylogeny. Nature 441, 77–80. (10.1038/nature04563) 16672968

[57] Zhu M, Wang W, Yu X 2010 *Meemannia eos*, a basal sarcopterygian fish from the Lower Devonian of China—expanded description and significance. In Morphology, phylogeny and paleobiogeography of fossil fishes (eds Elliot DK, Maisey JG, Yu K, Miao D), pp. 199–214. Munich, Germany: Verlag, Dr. Friedrich Pfeil.

[58] Sallan LC, Friedman M 2011 Heads or tails: staged diversification in vertebrate evolutionary radiations. Proc. R. Soc. B 279, 2025–2032. (10.1098/rspb.2011.2454) PMC3311904 22189401

[59] Friedman M, Sallan LC 2012 Five hundred million years of extinction and recovery: a Phanerozoic survey of large-scale diversity patterns in fishes. Palaeontology 55, 707–742. (10.1111/j.1475-4983.2012.01165.x)

[60] Huxley TH 1861 Preliminary essay upon the systematic arrangement of the fishes of the Devonian Epoch. Mem. Geol. Surv. UK Decade 10, 1–40.

[61] Cope ED 1871 Contribution to the ichthyology of the Lesser Antilles. Trans. Am. Phil. Soc. 14, 445–483. (10.2307/1005256)

[62] Gardiner BG 1963 Certain palaeoniscoid fishes and the evolution of the snout in actinopterygians. Bull. Br. Mus. Nat. Hist. 8, 258–325.

[63] Pradel A, Maisey JG, Tafforeau P, Mapes RH, Mallatt J 2014 A Palaeozoic shark with osteichthyan-like branchial arches. Nature 509, 608–611. (10.1038/nature13195) 24739974

[64] Jarvik E 1980 Basic structure and evolution of vertebrates, vol 1 London, UK: Academic Press.

[65] Chang M-M 1982 The braincase of *Youngolepis*, a Lower Devonian crossopterygian from Yunnan, South-Western China. PhD thesis, University of Stockholm, Sweden.

