# Supplementary material for: An exceptionally preserved Late Devonian actinopterygian provides a new model for primitive cranial anatomy in ray-finned fishes
Source: Proc Biol Sci. 2015 Oct 7;282(1816):20151485. doi: 10.1098/rspb.2015.1485 (PMC4614771; doi:10.1098/rspb.2015.1485)
Supplement: Supplementary Notes [file rspb20151485supp1.docx]

­Supplementary Notes

**Table of Contents**

Part A. Supplementary Notes

1. Geological Settings

Part B. Phylogenetic Analyses

1. Character List
2. List of Taxa

Part C. Supplementary References

**Part A. Supplementary Notes**

**1) Geological Setting**

The specimen described in this paper (MGL 1245) was collected by Mr Cristian Loonesfrom the now-flooded La Parisienne quarry, from the upper part of the Grey Member of the Ferques Formation, Ferques inlier, Pas-de-Calais, France. Preliminary descriptions were published by Darras (2006) and Murray (2012). Darras (2006) identified the specimen as an actinopterygian and briefly described the anatomy visible on the external surfaces, noting many similarities to *Mimipiscis* and *Moythomasia* (Gardiner, 1984). A preliminary low resolution scan was worked on by Murray (2012), who identified the presence of a complete braincase and some elements of the gill skeleton. An attempt at description was made, but given the low resolution of the scan only major features of the braincase and dermal skeleton could be described. Actinopt microremains, including scales and teeth, have also been collected from the Ferques region by a Palaeontological Association field meeting, and were described by Derycke et al. (1995). The Ferques Formation is associated with the lower *Ancyrognathus triangularis* conodont zone (Brice, 1988: fig. 2, pp. 15; Derycke et al., 1995: fig. 6), equivalent to the Frasnian late *Palmatolepis* *hassi* conodont zone of the Standard Conodont Zonation in Ziegler & Sandberg (1990: fig. 2; 2000: fig. 1) and Derycke-Khatir (2005: fig. 56). The late *hassi* zone corresponds to ~373 Ma (Gradstein et al., 2012: fig. 22.10).

Three of the scales (CVUL 38, CVUL 39, CVUL 40) were referred to *Moythomasia* sp., with a further scale (CVUL 44), a hemilepidotrichium (CVUL 46), a toothplate (CVUL 41) and an isolated tooth (CVUL 42) referred to Actinopterygii gen. et sp. indet. Additional material found in the Devonian of the Ferques region includes *Rhynchodus*? sp., *Symmorium*? sp., *Ctenacanthus* sp., Chondrichthyes gen. et sp. indet., *Ptomacanthus*? sp., *Acanthodes* sp., Acanthodii gen. et sp. indet., Onychodontida gen. et sp. indet., Porolepiformes gen. et sp. indet. and *Spodichthys*? sp.

Specimens CVUL 38, CVUL 39, CVUL 40 were referred to *Moythomasia* sp. due to similarities between them and those described by Jessen (1968). However, the scales of *Raynerius splendens* (Supplementary Fig. 1) also bear clear similarities with those of *Moythomasia*, most notably in the angled ganoin ridges and presence of open pores. As such, the scales described by Derycke et al. (1995) may well belong to *Raynerius splendens* but due to a lack a clear association are not currently referred to the new genus.

**Part B. Phylogenetic Analyses**

**1) Character List**

The character list is modified from that of Choo (2011), with substantial modifications in order to expand the analysis outside of actinopterygians. A number of characters have been deleted from Choo’s (2011) matrix, and justification for these deletions is given after the character list. Ordered characters are indicated by an asterisk (‘*’).

**1.** **Large dermal plates:**

Forey, 1980; Gardiner, 1984; Zhu & Schultze, 2001; Zhu et al., 2001; Zhu & Yu, 2002; Zhu et al., 2006; Friedman, 2007; Brazeau, 2009; Zhu et al., 2009; Friedman & Brazeau; 2010; Davis et al., 2012; Zhu et al., 2013; Brazeau & Friedman, 2014; Giles et al., 2015b.

0. absent

1. present

**2.** **Sensory lines:**

Brazeau, 2009; Zhu et al., 2013; Giles et al., 2015b.

0. preserved as open grooves

1. pass through canals

**3.** **[CH 1] Premaxillae, contact at midline:**

Cloutier & Ahlberg, 1996; Taverne, 1997; Schultze & Cumbaa, 2001; Zhu & Schultze, 2001; Zhu & Yu, 2002; Cloutier & Arratia, 2004; Friedman & Blom, 2006; Zhu et al., 2006; Friedman, 2007; Long et al., 2008; Swartz, 2009; Choo, 2011. The premaxillae in *Mansfieldiscus* (Long, 1988) are thought to have contacted at the midline but have not been observed, and so the coding for this taxon is conservatively changed from '0' to ‘?’. The snout is not preserved in *Novagonatodus* (Long, 1988; Holland et al., 2007), so the coding is changed from ‘1’ to ‘?’. Although a median dentigerous 'rostral' is preserved in *Luederia* (Schaeffer & Dalquest, 1978), the lack of associated bones means its identity is uncertain, and it may well represent fused premaxillae. This taxon is conservatively coded ‘?’. The premaxilla is absent in *Wendichthys* (Lund & Poplin, 1997), so the coding is revised from '0' to '-'. The coding for *Moythomasia lineata* is revised to '0' (Choo, 2015).

0. present

1. absent

**4.** **Premaxilla:**

Friedman, 2007; Giles et al., 2015b. The coding for *Moythomasia lineata* is revised to '0' (Choo, 2015).

0. Reaches or extends past anterior margin of orbit

1. Confined to region anterior to orbit

**5.** **Premaxilla contributes to orbital margin:**

Cloutier & Ahlberg, 1996; Schultze & Cumbaa, 2001; Zhu & Schultze, 2001; Zhu et al., 2001; Zhu & Yu, 2002; Cloutier & Arratia, 2004; Zhu et al., 2006; Friedman, 2007; Long et al., 2008; Swartz, 2009; Zhu et al., 2009; Xu & Gao, 2011; Zhu et al., 2013; Xu et al., 2014. This character is coded as inapplicable in taxa where the premaxilla is restricted anterior to the orbit. The coding for *Moythomasia lineata* is revised to '1' (Choo, 2015).

0. absent

1. present

**6.** **[CH 3] Postrostrals (element[s] immediately anterior to frontals but not in contact with premaxillae):**

Cloutier & Ahlberg, 1996; Taverne, 1997; Lund, 2000; Schultze & Cumbaa, 2001; Zhu & Schultze, 2001; Lund & Poplin, 2002; Cloutier & Arratia, 2004; Friedman & Blom, 2006; Long et al., 2008; Swartz, 2009; Choo, 2011; Xu et al., 2014. Choo's (2011) codes for this character appear reversed.

0. present

1. absent

**7.** **[CH 4] Single median dermal bone capping snout:**

Gardiner & Schaeffer, 1989; Taverne, 1997; Friedman & Blom, 2006; Long et al., 2008; Swartz, 2009; Choo, 2011.

0. absent

1. present

**8.** **Pores for rostral organ:**

Friedman, 2007.

0. absent

1. present

**9.** **[CH 5] Transverse ornamentation of median rostral or dermal cover of ethmoid region:**

Friedman & Blom, 2006; Long et al., 2008; Swartz, 2009; Choo, 2011. The ornament in *Osorioichthys* (Taverne, 1997) is not preserved on the posterior portion of the rostral in sufficient detail to code this character (pers. obs. S.G.). As such, the coding is changed from ‘0’ to ‘?’.

0. absent

1. present

**10.** **[CH 8] Nasal bone as single consolidated ossification (i.e. bone(s) carrying supraorbital canal between premaxilla and anterior margin of frontals):**

Taverne, 1997; Schultze & Cumbaa, 2001; Friedman & Blom, 2006; Long et al., 2008; Swartz, 2009; Choo, 2011.

0. absent

1. present

**11.** **[CH 57] Mesial margin of (anterior) nasal:**

Lund et al., 1995; Ahlberg & Johanson, 1998; Ahlberg et al., 2000; Lund, 2000; Poplin & Lund, 2000; Schultze & Cumbaa, 2001; Lund & Poplin, 2002; Cloutier & Arratia, 2004; Zhu & Ahlberg, 2004; Daeschler et al., 2006; Long et al., 2006; Zhu et al., 2006; Zhu et al., 2009; Choo, 2011. The nasal is poorly preserved in *Cheirolepis* *canadensis* (Pearson & Westoll, 1979; Arratia & Cloutier, 1996), and coding for this taxon is revised from ‘0’ to ‘?’. The position of the nostrils is not clear in *Tegeolepis*, and the coding for this taxon is revised from '1' to '?'.

0. not notched

1. notched

**12.** **[CH 6] Posterior nostril in complete communication with orbital fenestra:**

Friedman & Blom, 2006; Long et al., 2008; Choo, 2011. The position of the nostrils is not clear in *Tegeolepis*, and the coding for this taxon is revised from '0' to '?'.

0. absent

1. present

**13.** **[CH 7] Posterior nostril – contribution to margin by premaxillae:**

Friedman & Blom, 2006; Long et al., 2008; Choo, 2011. The coding for *Howqualepis* *rostridens* (Long, 1988) and *Gogosardina* (Choo et al., 2009) is revised from ‘0’ to ‘1’. The premaxilla is not preserved in *Novagonatodus* (Long, 1988; Holland et al., 2007), so the coding is changed from ‘0’ to ‘?’. The premaxilla is absent in *Wendichthys* (Lund & Poplin, 1997), so the coding is revised from '0' to '-'. The position of the nostrils is not clear in *Tegeolepis*, and the coding for this taxon is revised from '0' to '?'. The coding for *Moythomasia lineata* is revised to '0' (Choo, 2015).

0. absent

1. present

**14.** **Tectals (sensu Cloutier & Ahlberg 1996, not counting the posterior tectal of Jarvik):**

Lund et al., 1995; Cloutier & Ahlberg, 1996; Lund, 2000; Schultze & Cumbaa, 2001; Zhu & Schultze, 2001; Zhu et al., 2001; Lund & Poplin, 2002; Zhu & Yu, 2002; Cloutier & Arratia, 2004; Zhu et al., 2006; Friedman, 2007; Swartz, 2009; Zhu et al., 2009; Zhu et al., 2013.

0. absent

1. present

**15.** **[CH 9] Pineal foramen:**

Cloutier & Ahlberg, 1996; Taverne, 1997; Schultze & Cumbaa, 2001; Zhu & Schultze, 2001; Zhu & Yu, 2002; Friedman & Blom, 2006; Friedman, 2007; Long et al., 2008; Brazeau, 2009; Swartz, 2009; Davis et al., 2012; Zhu et al., 2013; Xu et al., 2014; Giles et al., 2015b. A pineal foramen is variably present in *Cheirolepis canadensis* (Pearson & Westoll, 1979; Arratia & Cloutier, 1996), C. trailli (Pearson & Westoll, 1979), *Kentuckia deani* (Rayner, 1951) and *Meemannia* (Zhu et al., 2010), and these taxa are coded '0/1' to reflect this polymorphism. The coding for *Moythomasia lineata* is revised to '0' (Choo, 2015).

0. present

1. absent

**16.** **Pineal eminence:**

Friedman, 2007; Zhu et al., 2009. Can only be coded in taxa that lack a pineal foramen.

0. absent

1. present

**17.** **[CH 10] Shape of parietals (sarcopterygian postparietals):**

Dietze, 2000; Schultze & Cumbaa, 2001; Cloutier & Arratia, 2004; Friedman & Blom, 2006; Long et al., 2008; Swartz, 2009; Choo, 2011; Xu et al., 2014. The coding for *Cuneognathus* (Friedman & Blom, 2006), *Kentuckia* *hlavini* (Dunkle, 1964) and *Stegotrachelus* (Swartz, 2009) is revised from ‘0’ to ‘1’. The coding for *Melanecta* (Coates, 1998) is revised from ‘1’ to ‘0’. *Wendichthys* (Lund & Poplin, 1997) was erroneously coded by Choo (2011) as state ‘2’, for which there is no description, and is recoded here as ‘1’. The coding for *Moythomasia lineata* is revised to '0' (Choo, 2015).

0. rectangular, with long axis parallel to midline

1. quadrate

**18.** **[CH 11] Relative lengths of frontals and parietals (sarcopterygian parietals and postparietals):**

Lund et al., 1995; Taverne, 1997; Dietze, 2000; Lund, 2000; Poplin & Lund, 2000; Schultze & Cumbaa, 2001; Lund & Poplin, 2002; Cloutier & Arratia, 2004; Friedman & Blom, 2006; Zhu et al., 2006; Long et al., 2008; Swartz, 2009; Choo, 2011; Xu et al., 2014. The coding for *Mimipiscis* *toombsi* (Gardiner, 1984; Choo, 2011) is changed from ‘1’ to ‘2’. The coding for *Moythomasia lineata* is revised to '2' (Choo, 2015).

0. frontal shorter than parietal

1. frontal approximately equal to parietal

2. frontal longer than parietal

**19.** **Anterior pit line:**

Giles et al., 2015b. Although not figured, an anterior pit line is described for *Miguashaia* (Cloutier 1996).

0. absent

1. present

**20.** **Otic canal extends through parietals:**

0. absent

1. present

**21.** **Tabular:**

Lund et al., 1995; Cloutier & Ahlberg, 1996; Schultze & Cumbaa, 2001; Zhu & Schultze, 2001; Cloutier & Arratia, 2004; Long et al., 2008; Swartz, 2009.

0. present

1. absent

**22.** **Tabular pit line:**

0. absent

1. present

**23.** **[CH 12] Intertemporal – relative length:**

Taverne, 1997; Friedman & Blom, 2006; Choo, 2011. The coding for *Moythomasia durgaringa* (Gardiner, 1984) and *Moythomasia nitida* (Jessen, 1968) is revised from '1' to '0'. The intertemporal appears to be absent in *Cheirolepis schultzei* (Arratia & Cloutier, 2004), so is coded '-'. The coding for *Moythomasia lineata* is revised to '0' (Choo, 2015).

0. shorter than supratemporal

1. of similar length to supratemporal

2. longer than supratemporal

**24.** **[CH 13] Intertemporal – contact with supratemporal anterior to that between frontal and parietal:**

Friedman & Blom, 2006; Choo, 2011. The coding for *Moythomasia lineata* is revised to '0' (Choo, 2015).

0. absent

1. present

**25.** **[CH 54] Dermosphenotic with distinct posterior ramus:**

Gardiner & Schaeffer, 1989; Coates, 1998; Schultze & Cumbaa, 2001; Cloutier & Arratia, 2004; Friedman & Blom, 2006; Zhu et al., 2006; Long et al., 2008; Zhu et al., 2009; Choo, 2011. The dermosphenotic illustrated by (Gardiner 1984, fig 69) lacks a posterior limb, but this is from a small individual and most likely reflects ontogenetic variability, with a posterior limb being developed in larger individuals (B. Choo, pers. comm.; Choo 2015: fig. 8). The shape of the dermosphenotic in *Cuneognathus* (Friedman & Blom, 2006) is inferred, and the coding is thus revised from ‘1’ to ‘?’. The coding in *Melanecta* (Coates, 1998) is revised from ‘0’ to ‘?’. The posterior limb of the dermosphenotic is variably developed in *Mesopoma* (Coates, 1999), so this taxon is scored '0/1' to reflect this polymorphism.

0. absent

1. present

**26.** **[CH 14] Dermosphenotic – contact with frontals blocked by intertemporal or dermopterotic:**

Friedman & Blom, 2006; Choo, 2011. The coding for *Moythomasia lineata* is revised to '0' (Choo, 2015).

0. absent

1. present

**27.** **Intertemporal contacts nasal:**

Xu & Gao, 2011; Xu et al., 2014.

0. absent

1. present

**28.** **[CH 64] Number of bones carrying supraorbital canal between dermosphenotic and posterior edge of skull roof. :**

Cloutier & Arratia, 2004; Choo, 2011. This character is reformulated from Choo's character 'Dermopterotic: present/absent'. The coding in *Melanecta* (Coates, 1998) has been revised from '0' to '1'. The coding in *Moythomasia* *nitida* (Jessen, 1968) has been revised from '1' to '0'. The coding for *Moythomasia lineata* is revised to '0' (Choo, 2015).

0. at least two (i.e. intertemporal and supratemporal)

1. one (i.e. dermopterotic)

**29.** **[CH 69] Supratemporal – narrow anterolateral flange forming ventral margin of spiracular opening:**

Choo, 2011. The coding for *Mimipiscis bartrami* and *M. toombsi* (Gardiner, 1984; Choo, 2011) is revised from ‘0’ to ‘1’. The coding for *Moythomasia* *durgaringa* (Gardiner, 1984) and *Moy. nitida* (Jessen, 1968) is revised from ‘1’ to ‘0’. The position of the spiracular space in *Stegotrachelus* is uncertain, so this taxon is coded as ‘?’. The posterior and ventral borders of the supratemporal are poorly preserved in *Krasnoyarichthys* (Prokofiev, 2002), so the coding is changed from ‘0’ to ‘?’. The coding for *Moythomasia lineata* is revised to '0' (Choo, 2015).

0. absent

1. present

**30.** **[CH 15] Number of paired extrascapulars:**

Gardiner & Schaeffer, 1989; Lund et al., 1995; Cloutier & Ahlberg, 1996; Coates, 1998; Lund, 2000; Poplin & Lund, 2000; Schultze & Cumbaa, 2001; Zhu & Schultze, 2001; Lund & Poplin, 2002; Cloutier & Arratia, 2004; Friedman & Blom, 2006; Long et al., 2008; Swartz, 2009; Choo, 2011; Zhu et al., 2013. The coding for *Moythomasia lineata* is revised to '0' (Choo, 2015).

0. one pair

1. two pairs

**31.** **Extrascapular reaches lateral edge of skull roof:**

New character. A complete skull roof of *Moythomasia durgaringa* is not figured by Gardiner (1984), but on the basis of his description (Gardiner, 1984: p. 318) it can be inferred that the extrascapular was blocked from reaching the lateral margin of the skull roof by the posterior flange of the supratemporal. This taxon is coded ‘0’. The extrascapular in *Cuneognathus* is incomplete laterally. However, the extensive posterolateral extension of the supratemporal (Friedman & Blom, 2006: fig. 3) makes it unlikely that the extrascapular would have reached the lateral edge of the skull roof, and as such this character is coded ‘0’. This convention is also followed for *Tegeolepis* (Dunkle, 1964), and *Meemannia* (Zhu et al., 2006). The coding for *Moythomasia lineata* is revised to '0' (Choo, 2015).

0. absent

1. present

**32.** **[CH 71] Single median extrascapular:**

Dietze, 2000; Cloutier & Arratia, 2004; Long et al., 2008; Swartz, 2009; Choo, 2011; Xu & Gao, 2011; Zhu et al., 2013; Xu et al., 2014. The coding in *Mimipiscis bartrami*, *M. toombsi* (Gardiner, 1984; Choo, 2011), *Stegotrachelus* (Swartz, 2009), *Cheirolepis canadensis* (Arratia & Cloutier, 1996), *C. schultzei* (Arratia & Cloutier, 2004), *C. trailli* (Pearson & Westoll, 1979), *Donnrosenia* (Long et al., 2008), *Howqualepis* (Long, 1988), Mansfieldiscus (Long, 1988) and *Woodichthys* (Coates, 1998) is revised from ‘0’ to ‘1’. The extrascapulars are not preserved in *Gogosardina* (Choo et al., 2009) and *Melanecta* (Coates, 1998), and the coding for these taxa is revised from ‘0’ to ‘?’. The median extrascapular in *Coccocephalichthys* has an anterior and posterior series, as well as being paired about the midline, and is coded as ‘0’ here. The coding for *Moythomasia lineata* is revised to '1' (Choo, 2015).

0. present

1. absent

**33.** **Extrascapulae contact each other at midline:**

New character. This character is coded as inapplicable for taxa that possess a median extrascapular, as it is logically impossible for the lateral extrascapulae to meet in the midline. It is unclear whether the extrascapulae met at the midline in *Moythomasia durgaringa* (Gardiner, 1984) or *Cuneognathus* (Friedman & Blom, 2006), so these taxa are coded '?'.

0. absent

1. present

**34.** **[CH 70] Medially-directed branch of sensory canal in extrascapulae:**

Choo, 2011. The codings for *Mimipiscis bartrami*, *M. toombsi* (Gardiner, 1984; Choo, 2011), *Osorioichthys* (Taverne, 1997), *Cheirolepis canadensis* (Arratia & Cloutier, 1996), *C. trailli* (Pearson & Westoll, 1979), *Mansfieldiscus* (Long, 1988), *Woodichthys* (Coates, 1998) and *Wendichthys* (Lund & Poplin, 1997) are revised from ‘1’ to ‘0’. Although often figured as present in reconstructions, it is unclear whether these canals were present in *Howqualepis rostridens* (Long, 1988), *Cuneognathus* (Friedman & Blom, 2006), *Donnrosenia* (Long et al., 2008), *Kentuckia hlavini* (Dunkle, 1964), *Limnomis* (Daeschler, 2000), *Stegotrachelus* (Swartz, 2009) and *Krasnoyarichthys* (Prokofiev, 2002), and the coding for these taxa is revised from ‘1’ to ‘?’. The coding for *Moythomasia lineata* is revised to '1' (Choo, 2015).

0. present

1. absent

**35.** **Extratemporal :**

Cloutier & Ahlberg, 1996; Ahlberg & Johanson, 1998; Zhu & Schultze, 2001; Zhu et al., 2001; Zhu & Yu, 2002; Zhu & Ahlberg, 2004; Daeschler et al., 2006; Long et al., 2006; Zhu et al., 2006; Friedman, 2007; Zhu et al., 2009.

0. absent

1. present

**36.** **[CH 75] Dermal intracranial joint:**

Cloutier & Ahlberg, 1996; Ahlberg & Johanson, 1998; Zhu & Ahlberg, 2004; Zhu & Schultze, 2001; Zhu et al., 2001; Zhu & Yu, 2002; Daeschler et al., 2006; Long et al., 2006; Zhu et al., 2006; Friedman, 2007; Brazeau, 2009; Zhu et al., 2009; Choo, 2011; Davis et al., 2012; Zhu et al., 2013. Choo’s coding for this character appears to be inaccurate, and codes have been changed where appropriate.

0. absent

1. present

**37.** **[CH 60] Suborbital bones contributing to dorsal margin of orbit:**

Choo, 2011. The coding for *Moythomasia lineata* is revised to '0' (Choo, 2015).

0. absent

1. present

**38.** **[CH 59] Antorbital bone:**

Cloutier & Arratia, 2004; Choo, 2011; Xu & Gao, 2011; Xu et al., 2014. The coding for *Moythomasia lineata* is revised to '0' (Choo, 2015).

0. absent

1. present

**39.** **[CH 61] Infraorbitals:**

Cloutier & Arratia, 2004; Gardiner et al., 2005; Choo, 2011; Xu & Gao, 2011; Xu et al., 2014. Choo's (2011) codes for this character appear reversed. The coding for *Gogosardina* (Choo et al., 2009) is revised from '0' to '?'. The coding for *Limnomis* (Daeschler, 2000) is revised from '?' to '0'. The coding for *Tegeolepis* (Dunkle & Schaeffer, 1973) is revised from '0' 'to '?'. The coding for *Moythomasia lineata* is revised to '0' (Choo, 2015).

0. two

1. more than two

**40.** **[CH 16] Anterior expansion of lacrimal:**

Taverne, 1997; Friedman & Blom, 2006; Long et al., 2008; Swartz, 2009; Choo, 2011. The coding for *Melanecta* (Coates, 1998) is revised from ‘0’ to ‘?’. The coding for *Miguashaia* (Cloutier, 1996) is revised from '1' to '?'.The coding for *Novagonatodus* (Long, 1988; Holland et al., 2007) and *Onychodus* (Andrews et al., 2006) is revised from ‘?’ to ‘1’. The coding for *Tegeolepis* (Dunkle & Schaeffer, 1973) is revised from '1' to '0'. The coding for *Wendichthys* (Lund & Poplin, 1997) is revised from '0' to '1'.

0. absent

1. present

**41.** **[CH 17] Notch in anterior margin of jugal:**

Cloutier & Arratia, 2004; Friedman & Blom, 2006; Long et al., 2008; Swartz, 2009; Choo, 2011; Xu et al., 2014. Although the jugal of *Moythomasia durgaringa* is only faintly notched in its reconstruction (Gardiner, 1984: fig. 103), the notch is clearly visible on the medial face (Gardiner, 1984: fig. 73). As such the coding is revised from ‘0’ to ‘1’. The coding in *Cuneognathus* (Friedman & Blom, 2006) is revised from’0’ to ‘?’. The coding in *Novagonatodus* (Long, 1988; Holland et al., 2007) is changed from ‘1’ to ‘0’.The coding in *Wendichthys* (Lund & Poplin, 1997) is revised from '1' to '0'. The coding for *Moythomasia lineata* is revised to '0' (Choo, 2015).

0. absent

1. present

**42.** **[CH 18] Jugal and maxilla separated by non-canal bearing ossificatons (i.e. anamestic suborbitals):**

Taverne, 1997; Schultze & Cumbaa, 2001; Friedman & Blom, 2006; Long et al., 2008; Choo, 2011.

0. absent

1. present

**43.** **Multiple rami of infraorbital canal in jugal:**

New character. Multiple branches radiate from the infraorbital canal in the jugal of many Carboniferous actinopts.

0. absent

1. present

**44.** **[CH 62] Jugal canal:**

Patterson, 1982; Lauder & Liem, 1983; Gardiner, 1984; Cloutier & Arratia, 2004; Brazeau, 2009; Friedman & Brazeau, 2010; Choo, 2011; Davis et al., 2012; Zhu et al., 2013; Giles et al., 2015b. Choo's (2011) codes may be reversed for this character, but it is unclear. The coding for *Cuneognathus* (Friedman & Blom, 2006), *Kentuckia hlavini*, *Krasnoyarichthys* and *Limnomis* is revised from '1' to ?'. The coding for *Donnrosenia*, *Gogosardina*, *Howqualepis*, *Masnfieldiscus*, *Melanecta*, *Mimipiscis bartrami*, *M. toombsi, Moythomasia durgaringa, Moy. nitida, Novagonatodus, Stegotrachelus* and *Woodichthys* is revisied from '1' to '0'. The coding for *Onychodus* is revised from '0' to '1'. The coding for *Miguashaia* is revised from '-' to '1'. The coding for *Moythomasia lineata* is revised to '0' (Choo, 2015).

0. absent

1. present

**45.** **[CH 53] Dermohyal:**

Patterson, 1982; Gardiner & Schaeffer, 1989; Lund et al., 1995; Cloutier & Ahlberg, 1996; Coates, 1998; Dietze, 2000; Lund, 2000; Schultze & Cumbaa, 2001; Zhu & Schultze, 2001; Zhu et al., 2001; Lund & Poplin, 2002; Zhu & Yu, 2002; Cloutier & Arratia, 2004; Gardiner et al., 2005; Friedman & Blom, 2006; Zhu et al., 2006; Friedman, 2007; Long et al., 2008; Swartz, 2009; Zhu et al., 2009; Choo, 2011; Xu & Gao, 2011; Xu et al., 2014. This region of the cheek is missing in *Coccocephalichthys* (Poplin, 1974; Poplin & Véran, 1996), and was presumably removed by Watson (1925) when he first described the specimen. It is unclear from the surviving cast whether a dermohyal and/or accessory operculum were present, and as such this taxon is coded as ‘?’. The presence of a dermohyal is only inferred in *Donnrosenia* (Long et al., 2008), and the coding for this taxon is revised from ‘1’ to ‘?'.

0. absent

1. present

**46.** **Head of dermohyal projects above dorsal margin of operculum:**

New character. The dermohyal is not preserved in *Melanecta* (Coates, 1998), but it is clear from the surrounding bones that it would not have projected above the dorsal surface of the operculum.

0. absent

1. present

**47.** **Dermohyal:**

Gardiner et al., 2005; Coates, 1999; Xu & Gao, 2011; Xu et al., 2014. The relevant part of the cheek is not preserved in *Donnrosenia* (Long et al., 2008), so the coding for this taxon is changed from '1' to '?'.

0. fused to hyomandibular

1. separate from hyomandibular

**48.** **[CH 58] Quadratojugal as distinct ossification:**

Lund et al., 1995; Cloutier & Ahlberg, 1996; Dietze, 2000; Schultze & Cumbaa, 2001; Zhu & Schultze, 2001; Cloutier & Arratia, 2004; Long et al., 2008; Swartz, 2009; Choo, 2011.The presence of a quadratojugal in *Stegotrachelus* (Swartz, 2009), *Moythomasia nitida* (Jessen, 1968) and *Limnomis* (Daeschler, 2000) is uncertain, and the coding for these taxa is revised from ‘1’ to ‘?’. A distinct quadratojugal is absent in *Moy. nitida* and *Moy. lineata.* The coding in *Cheirolepis canadensis* (Arratia & Cloutier, 1996) and *Wendichthys* (Lund & Poplin, 1997) is revised from ‘1’ to ‘0’.

0. present

1. absent

**49.** **Complete enclosure of spiracle by bones bearing otic and infraorbital canals:**

Friedman, 2007; Zhu et al., 2009. The position of the spiracular space in *Stegotrachelus* (Swartz, 2009) is uncertain, so this taxon is coded as '?'. The coding for *Moythomasia lineata* is revised to '1' (Choo, 2015).

0. absent

1. present

**50.** **[CH 19] Accessory operculum:**

Schultze & Cumbaa, 2001; Cloutier & Arratia, 2004; Friedman & Blom, 2006; Long et al., 2008; Swartz, 2009. This region of the cheek was removed in *Coccocephalichthys* (Poplin, 1974; Poplin & Véran, 1996), presumably by Watson (1925) when he first described the specimen. It is unclear from the surviving cast whether a dermohyal and/or accessory operculum were present, and as such this taxon is coded as ‘?’.

0. absent

1. present

**51.** **[CH 67] Operculum - relative size :**

Lund et al., 1995; Lund, 2000; Lund & Poplin, 2002; Cloutier & Arratia, 2004; Long et al., 2008; Swartz, 2009; Choo, 2011. The coding in *Osorioichthys* (Taverne, 1997), *Mansfieldiscus* (Long, 1988), *Melanecta* (Coates, 1988), *Moythomasia nitida* (Jessen, 1968), *Novagonatodus* (Long, 1988; Holland et al., 2007), *Woodichthys* (Coates, 1998), *Cuneognathus* (Friedman & Blom, 1006) and *Krasnoyarichthys* (Prokofiev, 2002) is revised from ‘0’ to ‘1’. The coding in *Howqualepis* (Long, 1988), *Donnrosenia* (Long et al., 2008) and *Limnomis* (Daeschler, 2000) is revised from ‘1’ to ‘0’.

0. at least twice as high as suboperculum

1. less than twice as high as suboperculum

**52.** **[CH 68] Anterodorsal process of suboperculum:**

Long et al., 2008; Choo, 2011. The coding in *Howqualepis* (Long, 1988) and *Donnrosenia* (Long et al., 2008) is changed from '0' to '1'. The anterodorsal process is described as well developed in *Gogosardina* (Choo et al., 2009), and the coding for this taxon is revised from '?' to '1'.

0. absent

1. present

**53.** **[CH 72] Branchiostegal rays - dorsal-most in series:**

Lund et al., 1995; Cloutier & Arratia, 2004; Choo, 2011. The coding for *Mimipiscis bartrami, M. toombsi* (Gardiner, 1984, Choo, 2011), *Stegotrachelus* (Swartz, 2009), *Cheirolepis canadensis* (Arratia & Cloutier, 1996), *C. schulzei* (Arratia & Cloutier, 2004), *C. trailli* (Pearson & Westoll, 1979) *Donnrosenia* (Long et al., 2008), *Gogosardina* (Choo et al., 2009), *Howqualepis rostridens* (Long, 1988), *Novagonatodus* (Long, 1988; Holland et al., 2007), Mansfieldiscus (Long, 1988) and *Woodichthys* (Coates, 1998) is revised from ‘1’ to ‘0’. The coding for *Cuneognathus* (Friedman & Blom, 2006), *Kentuckia hlavini* (Dunkle, 1964) and *Melanecta* (Coates, 1998) is revised from ‘1’ to ‘?’. The coding for *Krasnoyarichthys* (Prokofiev, 2002) is changed from ‘?’ to ‘1’.

0. of similar depth to adjacent branchiostegal ray

1. deeper than adjacent branchiostegal ray

**54.** **[CH 20] Lateral gulars:**

Gardiner & Schaeffer, 1989; Cloutier & Ahlberg, 1996; Taverne, 1997; Lund & Poplin, 1997; Coates, 1999; Schultze & Cumbaa, 2001; Zhu & Schultze, 2001; Cloutier & Arratia, 2004; Friedman & Blom, 2006; Long et al., 2008; Swartz, 2009; Brazeau, 2009; Xu & Gao, 2011; Davis et al., 2012; Zhu et al., 2013; Xu et al., 2014; Giles et al., 2015b. The coding for *Mansfieldiscus* (Long, 1988) is revised from ‘?’ to ‘1’. The condition in *Boreosomus* (Nielsen, 1942) is unique: instead of lateral gulars flanking a median gular, there appears to be a second median gular. This may well represent a fusion of the two, longer lateral gulars, is coded as such. The coding for *Moythomasia lineata* is revised to '0' (Choo, 2015).

0. extending most of the length of the lower jaw

1. restricted to the anterior third of the lower jaw (no longer than the width of three branchiostegals)

**55.** **Median gular:**

Lund et al., 1995; Cloutier & Ahlberg, 1996; Coates, 1999; Lund, 2000; Schultze & Cumbaa, 2001; Zhu & Schultze, 2001; Zhu et al., 2001: Lund & Poplin, 2002; Zhu & Yu, 2002; Cloutier & Arratia, 2004; Zhu et al., 2006; Friedman, 2007; Zhu et al., 2009; Xu & Gao, 2011; Zhu et al., 2013; Xu et al., 2014; Giles et al., 2015b. Pearson & Westoll (1979: p. 365) state that a median gular is not known in *Cheirolepis canadensis*. Although a median gular is reconstructed by Cloutier & Arratia (1996: fig. 7), this bone is not present in any specimen photos and is not mentioned in the text. As such, this taxon is coded as ‘?’.

0. absent

1. present

**56.** **Relative length of median gular:**

New character. The condition in *Boreosomus* (Nielsen, 1942) is unique: instead of lateral gulars flanking a median gular, there appears to be a second median gular. This may well represent a fusion of the two, longer lateral gulars, and is coded as such.

0. shorter than lateral gulars

1. same length as or longer than lateral gulars

**57.** **Maxilla:**

Zhu & Yu, 2002; Friedman, 2007; Xu et al., 2014.

0. absent

1. present

**58.** **Expanded dorsal lamina of maxilla:**

Lund et al., 1995; Lund, 2000; Poplin & Lund, 2000; Schultze & Cumbaa, 2001; Zhu & Schultze, 2001; Zhu et al., 2001; Zhu & Yu, 2002; Lund & Poplin, 2002; Cloutier & Arratia, 2004; Zhu et al., 2006; Friedman, 2007; Zhu et al., 2009; Zhu et al., 2013; Giles et al., 2015b.

0. absent

1. present

**59.** **[CH 63]** **Maxilla - posteroventral overlap of lower jaw:**

Cloutier & Arratia, 2004; Choo, 2011.

0. posteroventral margin of maxilla largely straight with minimal overlap of lower jaw

1. posteroventral extension of maxilla with pronounced overlap of lower jaw

**60.** **Contribution by maxilla to posterior margin of cheek:**

Friedman, 2007; Zhu et al., 2009; Zhu et al., 2013; Giles et al., 2015b.

0. absent

1. present

**61.** **Sensory canal/pit line associated with maxilla:**

Friedman, 2007; Zhu et al., 2009; Zhu et al., 2013;

0. absent

1. present

**62.** **Number of cheek bones bearing pre-opercular canal posterior to jugal:**

Friedman, 2007; Zhu et al., 2009; Zhu et al., 2013. .

0. one

1. two

**63.** **[CH 21] Course of mandibular canal:**

Friedman & Blom, 2006; Long et al., 2008; Swartz, 2009; Choo, 2011. The coding for *Moythomasia lineata* is revised to '0' (Choo, 2015).

0. traces ventral margin of jaw along entire length

1. arches dorsally in anterior half of jaw

**64.** **Mandibular canal reaches anterior margin of mandible:**

New character. The mandibular canal is reconstructed as reaching the anterior margin of the dentary in *Cuneognathus* (Friedman & Blom, 2006), but specimen photos appear to show it leaving through the dorsal margin. As such, this taxon is coded '1' for this character.

0. present

1. absent

**65.** **[CH 74]** **Mandibular canal:**

Patterson, 1982; Cloutier & Ahlberg, 1996; Coates, 1998; Schultze & Cumbaa, 2001; Zhu & Schultze, 2001; Zhu et al., 2001; Zhu & Yu, 2002; Cloutier & Arratia, 2004; Zhu et al., 2006; Friedman, 2007; Zhu et al., 2009; Choo, 2011; Zhu et al., 2013. The coding for *Moythomasia lineata* is revised to '1' (Choo, 2015).

0. primarily carried by infradentaries

1. primarily carried by dentary

**66.** **Relative length of dentary:**

Ahlberg & Johanson, 1998; Zhu et al., 2001; Zhu & Yu, 2002; Zhu & Ahlberg, 2004; Friedman, 2007; Zhu et al., 2009.

0. long (constitutes most of the length of the lower jaw)

1. short (constitutes less than half of jaw length)

**67.** **[CH 22] Dentary with conspicuously reflexed distal tip:**

Friedman & Blom, 2006; Long et al., 2008; Swartz, 2009; Choo, 2011. The anterior extent of the dentary is not preserved in *Limnomis* (Daeschler, 2000), so the coding for this taxon is revised from '1' to '?'.

0. absent

1. present

**68.** **[CH 24] Enlarged series of parasymphysial teeth on dentary:**

Friedman & Blom, 2006; Long et al., 2008; Swartz, 2009; Choo, 2011. Choo's (2011) codes for this character appear reversed. The anterior extent of the dentary is not preserved in *Limnomis* (Daeschler, 2000), so the coding for this taxon is revised from '1' to '?'.

0. absent

1. present

**69.** **[CH 73] Facet for parasymphysial tooth-whorl on anterior dentary:**

Choo, 2011. The anterior extent of the dentary is not preserved in *Limnomis* (Daeschler, 2000), so the coding for this taxon is revised from '1' to '?'.

0. present

1. absent

**70.** **Teeth of outer dental arcade:**

Friedman, 2007. Long (1988) states that the maxilla of *Melanecta* bears large teeth interspersed with smaller teeth, but it is unclear how these teeth are arranged. As such, this taxon is coded '?'.

0. several rows of disorganized teeth

1. two rows, with large teeth lingually and small teeth labially

2. single row of teeth

**71.** **[CH 25] Acrodin caps on teeth:**

Patterson, 1982; Gardiner, 1984; Maisey, 1986; Gardiner & Schaeffer, 1989; Cloutier & Ahlberg, 1996; Taverne, 1997; Coates, 1999; Poplin & Lund, 2000; Schultze & Cumbaa, 2001; Zhu & Schultze, 2001; Zhu et al., 2001; Zhu & Yu, 2002; Cloutier & Arratia, 2004; Gardiner et al., 2005; Friedman & Blom, 2006; Zhu et al., 2006; Friedman, 2007; Long et al., 2008; Zhu et al., 2009; Friedman & Brazeau, 2010; Choo, 2011; Xu & Gao, 2011; Zhu et al., 2013; Xu et al., 2014; Giles et al., 2015b. The presence of acrodin in *Limnomis* (Daeschler, 2000), *Mansfieldiscus* (Long, 1988), *Melanecta* (Coates, 1998) and *Woodichthys* (Coates, 1998) is uncertain, and the coding for these taxa is revised from '1' to '?'.

0. absent

1. present

**72.** **[CH 26] Lower jaw ornament smooth and punctuated by regular large pores:**

Friedman & Blom, 2006; Long et al., 2008; Swartz, 2009; Choo, 2011. This character is reformulated from Choo’s character: Remodeled porous ganoine on lower jaw. Although the dermal ornament is poorly preserved in *Osorioichthys*, faint ridges are visible on the dentary (Takverne, 1997: p.67; pers. obs. S.G.). As such, this character is coded as '0'. The coding for *Moythomasia lineata* is revised to '0' (Choo, 2015).

0. absent

1. present

**73.** **[CH 27] Ossification of mentomeckelian region:**

Friedman & Blom, 2006; Long et al., 2008; Swartz, 2009; Choo, 2011; Xu et al., 2014.

0. present

1. absent

**74.** **Coronoids (sensu stricto, excluding parasymphysial tooth whorl or anterior coronoid):**

Schultze & Cumbaa, 2001; Zhu & Schultze, 2001; Zhu et al., 2001; Zhu & Yu, 2002; Zhu et al., 2006; Friedman, 2007; Zhu et al., 2009.

0. present

1. absent

**75.** **Number of coronoids*****:**

Ahlberg & Clack, 1998; Daeschler et al., 2006; Long et al., 2006; Friedman, 2007; Zhu et al., 2009; Zhu et al., 2013; Giles et al., 2015b. A single specimen of *Pteronisculus stensioi* has at least five or six coronoids anterior to the prearticular region. However, these appear to correspond to the three coronoids present in most specimens, so the taxon is coded here as '2'. Two coronoids are reported in *Boreosomus* (Nielsen, 1942).

0. five

1. four

2. three

3. two

**76.** **Posterior coronoid:**

Cloutier & Ahlberg, 1996; Ahlberg & Johanson, 1998; Zhu & Ahlberg, 2004; Daeschler et al., 2006; Long et al., 2006.

0. morphologically similar to anterior coronoids

1. expanded

**77.** **[CH 23] Number of infradentaries*****:**

Friedman & Blom, 2006; Friedman, 2007; Long et al., 2008; Choo, 2011; Xu & Gao, 2011; Xu et al., 2014. The coding for *Kentuckia hlavini* (Dunkle, 1964) and *Guiyu* (Zhu et al., 2009) is revised from '0' to '?'. The coding for *Limnomis* (Daeschler, 2000) and *Stegotrachelus* (Swartz, 2009) is revised from '1' to '?'. The coding in *Mansfieldiscus* (Long, 1988) is revised from '0' to '2'. The coding for *Moythomasia lineata* is revised to '1' (Choo, 2015).

0. more than two

1. two (angular and surangular)

2. one (angular only)

**78.** **Expanded crest on posterior infradentary:**

Zhu & Yu, 2002; Friedman, 2007.

0. absent

1. present

**79.** **Retroarticular process:**

Friedman, 2007.

0. present

1. absent

**80.** **Articular area for symplectic:**

Friedman, 2007.

0. present

1. absent

**81.** **Number of dermopalatines:**

Friedman, 2007.

0. multiple

1. single

**82.** **Basipterygoid fenestra :**

Friedman, 2007; Brazeau, 2009; Zhu et al., 2009; Friedman & Brazeau, 2010; Davis et al., 2012; Zhu et al., 2013; Giles et al., 2015b.

0. absent

1. present

**83.** **Fenestra ventrolateralis:**

Schultze & Cumbaa, 2001; Zhu & Schultze, 2001; Zhu et al., 2001; Zhu & Yu, 2002; Zhu et al., 2006; Friedman, 2007; Zhu et al., 2009; Zhu et al., 2013.

0. absent

1. present

**84.** **Palatal opening surrounded by premaxilla, maxilla, dermopalatine and vomer (choana):**

Zhu & Yu, 2002; Friedman, 2007. Character can only be coded in taxa that possess all of these bones.

0. absent

1. present

**85.** **Internasal cavity:**

Ahlberg & Johanson, 1998; Zhu & Yu, 2002; Zhu & Ahlberg, 2004; Daeschler et al., 2006; Long et al., 2006; Friedman, 2007; Zhu et al., 2009; Zhu et al., 2013; Giles et al., 2015b.

0. absent

1. present

**86.** **Interorbital septum:**

Friedman, 2007; Zhu et al., 2009; Brazeau, 2009; Friedman & Brazeau, 2010; Davis et al., 2012; Zhu et al., 2013; Giles et al., 2015b. *Cheirolepis trailli* is coded '0' (Giles et al., 2015a).

0. broad

1. narrow

**87.** **Pronounced median anterior crista on dorsal surface of braincase:**

New character. Carboniferous and younger actinopts such as *Lawrenciella* (Hamel & Poplin, 2008) have a median crista anterior to the anterior dorsal fontanelle upon which the skull roof sits.

0. absent

1. present

**88.** **Expanded anterior dorsal fontanelle:**

New character. The anterior dorsal fontanelle of many Carboniferous and younger actinopts is greatly expanded, in contrast to the smaller fontanelle of Devonian taxa such as *Mimipiscis* (Gardiner, 1984).

0. absent

1. present

**89.** **Endoskeletal intracranial joint:**

Cloutier & Ahlberg, 1996; Ahlberg & Johanson, 1998; Zhu & Ahlberg, 2004; Zhu et al., 2001; Zhu & Yu, 2002; Daeschler et al., 2006; Long et al., 2006; Friedman, 2007; Brazeau, 2009; Zhu et al., 2009; Friedman & Brazeau, 2010; Davis et al., 2013; Zhu et al., 2013; Giles et al., 2015b. *Cheirolepis trailli* is coded '0' (Giles et al., 2015a).

0. absent

1. present

**90.** **Eye stalk or unfinished area for similar structure:**

Zhu & Schultze, 2001; Zhu et al., 2001; Zhu & Yu, 2002; Zhu et al., 2006; Friedman, 2007; Zhu et al., 2009; Zhu et al., 2013; Giles et al., 2015b. This character is coded as absent for taxa that possess a large interorbital fenestra (e.g. *Pteronisculus, Coccocephalichthys, Kentuckia deani*), as, if present, the eyestalk area would be visible posterior to the opening for the optic nerve.

0. absent

1. present

**91.** **Roof of posterior myodome perforated by palatine branch of facial nerve (VII):**

Coates, 1999.

0. absent

1. present

**92.** **Foramen for abducens nerve (VI) dorsally positioned (level with optic foramen (II)):**

Coates, 1999.

0. absent

1. present

**93.** **Anterodorsal myodome:**

Coates, 1999; Xu & Gao, 2011; Xu et al., 2014.

0. paired

1. single

**94.** **Canal for pituitary vein:**

Coates, 1999; Xu et al., 2014.

0. present

1. enlarged

2. obliterated

**95.** **Pituitary vein foramen:**

Ahlberg & Johanson, 1998; Zhu & Ahlberg, 2004; Zhu & Schultze, 2001; Zhu et al., 2001; Zhu & Yu, 2002; Zhu et al., 2006; Friedman, 2007; Zhu et al., 2009; Davis et al., 2012; Zhu et al., 2013; Giles et al., 2015b.

0. dorsal to basipterygoid process

1. anterior to basipterygoid process

2. posterior to basipterygoid process

**96.** **Basicranial fenestra:**

Ahlberg & Johanson, 1998; Zhu et al., 2001; Zhu & Yu, 2002; Zhu & Ahlberg, 2004; Friedman, 2007; Zhu et al., 2009; Zhu et al., 2013; Giles et al., 2015b. *Cheirolepis trailli* is coded '0' (Giles et al., 2015a).

0. absent

1. present

**97.** **Spiracular groove on lateral commissure:**

Davis et al., 2012; Zhu et al., 2013; Giles et al., 2015b.

0. absent

1. present

**98.** **Spiracle housed in:**

Patterson, 1982; Gardiner, 1984; Gardiner & Schaeffer, 1989; Taverne, 1997; Coates, 1999; Gardiner et al., 2005; Xu & Gao, 2011; Xu et al., 2014. Taxa that lack a groove on the lateral commissure are coded as inapplicable for this character. Following Xu & Gao (2014), the spiracle in *Moythomasia durgaringa* is coded '1'. *Cheirolepis trailli* is coded '0' (Giles et al., 2015a).

0. groove

1. canal

**99.** **Spiracular grooves extending onto basicranial surface:**

Ahlberg & Johanson, 1998; Zhu & Ahlberg, 2004; Zhu & Schultze, 2001; Zhu et al., 2001; Zhu & Yu, 2002; Zhu et al., 2006; Daeschler et al., 2006; Long et al., 2006; Friedman, 2007; Brazeau, 2009; Zhu et al., 2009; Friedman & Brazeau, 2010; Davis et al., 2012; Zhu et al., 2013; Brazeau & Friedman, 2014; Giles et al., 2015b. *Cheirolepis trailli* is coded '1' (Giles et al., 2015a).

0. absent

1. present

**100.** **Basipterygoid process:**

Gardiner et al., 2005; Xu & Gao, 2011; Xu et al., 2014.

0. present

1. absent

**101.** **Basipterygoid process with vertically oriented component:**

Ahlberg & Johanson, 1998; Zhu & Schultze, 2001; Zhu et al., 2001; Zhu & Yu, 2002; Zhu & Ahlberg, 2004; Zhu et al., 2006; Friedman, 2007; Zhu et al., 2009; Davis et al., 2012; Zhu et al., 2013; Giles et al., 2015b.

0. absent

1. present

**102.** **Dermal component to basipterygoid process:**

Gardiner, 1984; Gardiner & Schaeffer, 1989; Taverne, 1997; Coates, 1999;

0. absent

1. present

**103.** **Prespiracular fossa:**

Poplin, 1984. *Cheirolepis trailli* is coded '0' (Giles et al., 2015a).

0. absent

1. present

**104.** **Fossa bridgei:**

Gardiner, 1984; Gardiner & Schaeffer, 1989; Taverne, 1997; Coates, 1999; Xu & Gao, 2011; Xu et al., 2014. Cheirolepis trailli is coded '0' (Giles et al., 2015a).

0. absent

1. present

**105.** **Posttemporal fossae:**

Zhu & Yu, 2002; Friedman, 2007.

0. absent

1. present

**106.** **Vestibular fontanelle:**

Friedman, 2007; Brazeau, 2009; Zhu et al., 2009; Friedman & Brazeau, 2010; Davis et al., 2012; Zhu et al., 2013; Brazeau & Friedman, 2014; Giles et al., 2015b. *Cheirolepis trailli* is coded '1' (Giles et al., 2015a).

0. absent

1. present

**107.** **Ventral cranial fissure and vestibular fontanelle:**

Coates, 1999. We follow Coates (1999) in coding *Howqualepis* as '0' on the basis of Long 1988 fig. 16 and AMF65495 (pers. obs. S.G.), rather than the braincase reconstruction (Long, 1988: fig. 18). *Cheirolepis trailli* is coded '0' (Giles et al., 2015a).

0. separated by bridge of bone

1. conflent

**108.** **Accessory fenestration in otic capsule :**

Friedman, 2007; Zhu et al., 2009. *Cheirolepis trailli* is coded '0' (Giles et al., 2015a).

0. absent

1. present

**109.** **Otoccipital fissure:**

Friedman, 2007; Brazeau, 2009; Davis et al., 2012; Zhu et al., 2013; Giles et al., 2015b. *Cheirolepis trailli* is coded '1' (Giles et al., 2015a).

0. absent

1. present

**110.** **Median projection overhanging posterior part of posterior dorsal fontanelle:**

New character. Variable in *Boreosomus*: the posterior dorsal fontanelle is closed in the specimen figured in Nielsen (1942: plate 25F), but developed in the specimen figured in plate 28. This taxon is coded ‘0/1’ to reflect this polymorphism.

0. absent

1. present

**111.** **Median projection overhanging anterior part of posterior dorsal fontanelle:**

New character. This projection is somewhat reduced in *Pteronisculus* (Nielsen, 1942), but is coded '1' here. Variable in *Boreosomus*: the posterior dorsal fontanelle is closed in the specimen figured in Nielsen 1942 plate 25F, but developed in the specimen figured in plate 28. This taxon is coded ‘0/1’ to reflect this polymorphism. *Cheirolepis trailli* is coded '0' (2015a).

0. absent

1. present

**112.** **Dorsal aorta*****:**

Coates & Sequeira, 1998; Coates & Sequeira, 2001a, b; Coates, 1999; Friedman, 2007; Zhu et al., 2009; Friedman & Brazeau, 2010; Zhu et al., 2013; Giles et al., 2015b. This character is coded as inapplicable in taxa that lack a canal for the dorsal aorta. *Cheirolepis trailli* is coded '0' (Giles et al., 2015a). The aortic canal of *Moythomasia* is not figured by Gardiner (1984), but a clear posterior notch in the aortic canal can be seen in Long & Trinajstic (2010:fig 5b). The neurocranium of *Gogosardina* is not yet described, but this character can be coded on the basis of Choo et al. (2009: fig. 9).

0. open in groove

1. canal notched posteriorly

2. completely enclosed in canal

**113.** **Dorsal aorta pierced by canal/s for exit of eff.a.2:**

New character. In *Mimipiscis bartrami* and *M. toombsi*, a groove for one of the efferent branchial arteries branches off from the lateral dorsal aorta immediately before the articular area for the first infrapharyngobranchial. However, it is uncertain which, so both taxa coded as '?' for these characters. The neurocranium of *Gogosardina* is not yet described, but this character can be coded on the basis of Choo et al. (2009: fig. 9).

0. absent

1. present

**114.** **Dorsal aorta pierced by canal/s for exit of eff.a.1:**

New character. In *Mimipiscis bartrami* and *M. toombsi*, a groove for one of the efferent branchial arteries branches off from the lateral dorsal aorta immediately before the articular area for the first infrapharyngobranchial. However, it is uncertain which, so both taxa coded as '?' for these characters. The neurocranium of *Gogosardina* is not yet described, but this character can be coded on the basis of Choo et al. (2009: fig. 9).

0. absent

1. present

**115.** **Bifurcation of dorsal aorta:**

Coates & Sequeira, 1998; Coates & Sequeira, 2001a, b; Coates, 1999; Friedman, 2007; Zhu et al., 2009; Friedman & Brazeau, 2010; Zhu et al., 2013; Giles et al., 2015b.

0. posterior to occiput

1. anterior to occiput

**116.** **Birfurcation of dorsal aorta into lateral dorsal aortae:**

Coates, 1999. This character is coded as inapplicable in taxa that lack a canal for the dorsal aorta. In *Mimipiscis toombsi*, the bifurcation point of the dorsal aorta can be open (Giles & Friedman, 2014: fig. 2) or closed (Gardiner 1984: fig. 15). This taxon is coded '0/1' to reflect this polymorphism. The aortic canal of *Moythomasia* is not figured by Gardiner (1984), but the bifucation into the lateral dorsal aortae can be seen in Long & Trinajstic (2010:fig 5b).

0. open

1. enclosed in canal

**117.** **Parasphenoid:**

Gardiner, 1984; Brazeau, 2009; Davis et al., 2012; Zhu et al., 2013; Giles et al., 2015b.

0. absent

1. present

**118.** **Parasphenoid:**

Coates, 1999; Zhu & Yu, 2002; Gardiner et al., 2005; Friedman, 2007, Xu & Gao, 2011; Xu et al., 2014. *Cheirolepis trailli* is coded '0' (Giles et al., 2015a).

0. terminates at/anterior to ventral otic fissure

1. extends across ventral otic fissure

**119.** **Parasphenoid protruding forwards into ethmoid region:**

Zhu & Schultze, 2001; Zhu et al., 2001; Zhu & Yu, 2002; Zhu et al., 2006; Friedman, 2007; Zhu et al., 2009; Zhu et al., 2013.

0. absent

1. present

**120.** **[CH 28] Ascending process of the parasphenoid:**

Patterson, 1982; Coates, 1999; Dietze, 2000; Schultze & Cumbaa, 2001; Zhu & Schultze, 2001; Cloutier & Arratia, 2004; Gardiner et al., 2005; Friedman & Blom, 2006; Zhu et al., 2006; Zhu et al., 2009; Choo, 2011; Xu & Gao, 2011; Zhu et al., 2013; Xu et al., 2014; Giles et al., 2015b. The coding in *Wendichthys* (Lund & Poplin, 1997) is revised from '?' to '1'.

0. absent

1. present

**121.** **[CH 29] Parasphenoid with multifid anterior margin:**

Friedman & Blom, 2006; Friedman, 2007; Zhu et al., 2009; Choo, 2011; Zhu et al., 2013; Giles et al., 2015b.

0. absent

1. present

**122.** **Parasphenoid denticle field:**

Friedman, 2007; Zhu et al., 2009; Zhu et al., 2013.

0. terminates at, or anterior to, level of internal carotid arteries

1. extends posterior to foramina for internal carotid arteries

**123.** **Denticulated field of parasphenoid:**

Ahlberg & Johanson, 1998; Zhu & Ahlberg, 2004; Zhu & Schultze, 2001; Zhu et al., 2001; Zhu & Yu, 2002; Daeschler et al., 2006; Zhu et al., 2006; Long et al., 2006; Friedman, 2007; Zhu et al., 2009; Zhu et al., 2013.

0. without spiracular groove

1. with spiracular groove

**124.** **Buccohypophyseal canal pierces parasphenoid:**

New character. The buccohypophyseal canal typically enters the dorsal surface of the parasphenoid, but whether it exits via the ventral surface is more variable, and this distribution is captured by this character.

0. present

1. absent

**125.** **Anterolaterally divergent olfactory tracts:**

Coates, 1999; Giles & Friedman, 2014. *Cheirolepis trailli* is coded '1' (Giles et al., 2015a).

0. absent

1. present

**126.** **Elongate olfactory tract(s:**

Brazeau, 2009; Friedman & Brazeau 2010; Davis et al., 2012; Zhu et al., 2013; Brazeau & Friedman, 2014; Giles & Friedman, 2014; Giles et al., 2015b. *Cheirolepis trailli* is coded '0' (Giles et al., 2015a). The olfactory tracts of *Osorioichthys* are elongate (pers. obs. unpubl. scan data S.G.).

0. absent

1. present

**127.** **Olfactory nerves carried in a single tract:**

Coates, 1999; Giles & Friedman, 2014. *Cheirolepis trailli* is coded '1' (Giles et al., 2015a). The olfactory nerves are carried in separate tracts in *Osorioichthys* (pers. obs. unpubl. scan data S.G.).

0. present

1. absent

**128.** **Hypophyseal chamber:**

Coates, 1999; Xu & Gao, 2011; Xu et al., 2014.

0. projects posteroventrally

1. projects ventrally or anteroventrally

**129.** **Optic lobes:**

Giles & Friedman, 2014.

0. narrower than cerebellum

1. same width or wider than cerebellum

**130.** **Optic tectum divided into bilateral halves:**

Coates, 1999.

0. absent

1. present

**131.** **Cerebellar corpus:**

New character. The region posterior to the cerebellar auricles in *Lawrenciella* was considered to be the area octavolateralis by Hamel & Poplin (2005). However, we interpret it as the corpus cerebellum (see Giles & Friedman, 2014), and this taxon is coded '1'.

0. absent

1. present

**132.** **Horizontal semicircular canal:**

Davis et al., 2012; Zhu et al., 2013; Giles & Friedman, 2014; Giles et al., 2015b.

0. joins vestibular region dorsal to ampulla for the posterior semicircular canal

1. joins vestibular region level with ampulla for the posterior semicircular canal

**133.** **Junction between ampulla of posterior semicircular canal and cranial cavity:**

New character. In certain primitive actinopts, such as *Mimipiscis* (Giles and Friedman, 2014), a short length of canal lies between the posterior ampulla and the remainder of the labyrinth.

0. separated by short length of canal

1. confluent

**134.** **Crus commune of anterior and posterior semicircular canal:**

Giles & Friedman, 2014.

0. dorsal to endocranial roof

1. ventral to endocranial roof

**135.** **Lateral cranial canal:**

Gardiner, 1984; Gardiner & Schaeffer, 1989; Coates, 1999; Cloutier & Arratia, 2004; Gardiner et al., 2005; Zhu et al., 2006; Zhu et al., 2009; Zhu et al., 2013; Giles & Friedman, 2014; Xu et al., 2014; Giles et al., 2015b. The presence of a lateral cranial canal in *Ligulalepis*, *Psarolepis* and *Meemannia* is uncertain. The fossae identified by Zhu et al. (2010) occupy a rather anterior position compared to the lateral cranial canal in actinopts, being situated lateral to the sacculus rather than posterior to it. As such, we regard the identity of these features as suspect, and these taxa are coded '?'.

0. absent

1. present

**136.** **Ceratohyal:**

Gardiner et al., 2005; Xu & Gao, 2011; Xu et al., 2014.

0. single ossification

1. two ossifications

**137.** **Anterior ossification of ceratohyal:**

Coates, 1999.

0. subcylindrical

1. hourglass-shaped

**138.** **Anterior ceratohyal:**

Coates, 1999. The groove for the afferent hyoidean artery in the ceratohyal of *Gogosardina* is visible in Choo et al. (2009: fig 5).

0. no groove

1. groove for afferent hyoidean artery

**139.** **Double headed hyomandibular:**

Cloutier & Ahlberg, 1996; Zhu & Schultze, 2001; Schultze & Cumbaa, 2001; Zhu et al., 2001; Zhu & Yu, 2002; Zhu et al., 2006; Friedman, 2007; Zhu et al., 2009; Friedman & Brazeau, 2010; Zhu et al., 2013; Giles et al., 2015b.

0. absent

1. present

**140.** **Perforate hyomandibula:**

Friedman, 2007; Zhu et al., 2009; Friedman & Brazeau, 2010; Xu & Gao, 2011; Zhu et al., 2013; Brazeau & Friedman, 2014; Xu et al., 2014. Although Long (1988: p.24) mentions the presence of a depression for the hyomandibular nerve in *Howqualepis*, it is unclear whether this perforated the hyomandibula. This taxon is conservatively coded as '?'. *Cheirolepis trailli* is coded '0' (Giles et al., 2015a). Following Friedman (2007), we code *Onychodus* as '0'.

0. absent

1. present

**141.** **Opercular process of hyomandibula:**

Gardiner & Schaeffer, 1989.

0. absent

1. present

**142.** **Endoskeletal urohyal:**

Friedman, 2007; Friedman & Brazeau, 2010; Giles et al., 2015b.

0. absent

1. present

**143.** **Basihyal:**

Davis et al., 2012; Zhu et al., 2013; Giles et al., 2015b.

0. absent

1. present

**144.** **Interhyal:**

Davis et al., 2012; Zhu et al., 2013; Giles et al., 2015b.

0. absent

1. present

**145.** **Hypohyal:**

Friedman & Brazeau, 2010; Brazeau & Friedman, 2014; Giles et al., 2015b.

0. absent

1. present

**146.** **Gill arches:**

Giles et al., 2015b.

0. largely restricted to area under braincase

1. extend far posterior to braincase

**147.** **[CH 30 in part] Enameloid on dermal bones and scales:**

Characters 147-150 form part of an atomisation of the compound characters 'ganoine' (typically defined as a single or multilayer enamel covering) and 'cosmine' (typically defined as a single layer of enamel with a well defined pore canal network) (e.g. Cloutier & Ahlberg, 1996; Ahlberg & Johanson, 1998; Zhu & Ahlberg, 2004; Schultze & Cumbaa, 2001; Zhu & Schultze, 2001; Zhu et al., 2001; Zhu & Yu, 2002; Daeschler et al., 2006; Long et al., 2006; Zhu et al., 2006; Zhu et al., 2009; Davis et al., 2012; Zhu et al., 2013). A similar approach to atomization was taken by Friedman (2007), Brazeau & Friedman (2010) and Giles et al. (2015b). As detailed histological investigations have not been carried out for the majority of early actinopterygians (rather, they have simply been described as being covered in/bearing ridges of ganoine), many of these characters cannot be coded for a number of taxa. Histological data are only known for specimens of *Mimipiscis toombsi* (Gardiner, 1984; Choo, 2011), so this and the following characters are coded '?' for *Mimipiscis bartrami* (Gardiner, 1984; Choo, 2011).

0. absent

1. present

**148.** **Extensive pore-canal network:**

See notes above for c. 147.

0. absent

1. present

**149.** **[CH 30 in part] Enamel:**

See notes above for c. 147.

0. single-layered

1. multi-layered

**150.** **[CH 30 in part] Enamel layers:**

See notes above for c. 147.

0. applied directly to one another

1. separated by layers of dentine

**151.** **[CH 34]** **Scales:**

Cloutier & Arratia, 2004; Friedman & Blom, 2006; Long et al., 2008; Swartz, 2009; Zhu et al., 2009; Choo, 2011.

0. micromeric

1. macromeric

**152.** **[CH 32 in part] Scales with ‘peg and socket articulation':**

Maisey, 1986; Gardiner & Schaeffer, 1989; Cloutier & Ahlberg, 1996; Coates, 1999; Dietze, 2000; Poplin & Lund, 2000; Schultze & Cumbaa, 2001; Cloutier & Arratia, 2004; Friedman & Blom, 2006; Friedman, 2007; Long et al., 2008; Brazeau, 2009; Swartz, 2009; Zhu et al., 2009; Friedman & Brazeau, 2010; Xu & Gao, 2011; Choo, 2011; Davis et al., 2012; Zhu et al., 2013; Xu et al., 2014; Giles et al., 2015b. This character is coded only for taxa that possess rhombic scales. The coding for *Kentuckia hlavini* (Dunkle, 1964) is revised from ‘1’ to ‘0’, and the coding for *Limnomis* (Daeschler, 2000) from ‘0’ to ‘1’. The coding for *Cheirolepis trailli* (Giles et al., 2015a) is revised from ‘0’ to ‘1’.

0. absent

1. present

**153.** **[CH 32 in part] Peg on rhomboid scale:**

Patterson, 1982; Cloutier & Ahlberg, 1996; Dietze, 2000; Schultze & Cumbaa, 2001; Zhu & Schultze, 2001; Zhu et al., 2001: Zhu & Yu, 2002; Cloutier & Arratia, 2004; Friedman & Blom, 2006; Zhu et al., 2006; Friedman, 2007; Zhu et al., 2009. Although peg-and-socket articulation of is present between the scales of *Limnomis* (Daeschler, 2000), the nature of the peg is not described. As such, this taxon is conservatively coded '?'. The coding for *Cheirolepis trailli* (Giles et al., 2015a) is revised from ‘?’ to ‘0’.

0. narrow

1. broad

**154.** **[CH 33] Anterodorsal process on scale:**

Patterson, 1982; Gardiner, 1984; Gardiner & Schaeffer, 1989; Schultze & Cumbaa, 2001; Zhu & Schultze, 2001; Zhu et al., 2001; Zhu & Yu, 2002; Cloutier & Arratia, 2004; Friedman & Blom, 2006; Zhu et al., 2006; Friedman, 2007; Long et al., 2008; Swartz, 2009; Zhu et al., 2009; Choo, 2011; Zhu et al., 2013; Giles et al., 2015b. The coding for *Limnomis* (Daeschler, 2000) and *Cheirolepis trailli* (Giles et al., 2015a) is revised from '0' to '1'.

0. absent

1. present

**155.** **[CH 35] Scales with well developed pores on surface:**

Friedman & Blom 2006; Long et al., 2008; Swartz, 2009; Choo, 2011; Xu et al., 2014. Scale crowns of *Cheirolepis schultzei* (Arratia & Cloutier, 2004) are not preserved, so this and the following scale characters are coded as '?'. The coding for *Donnrosenia* (Long et al., 2008) is revised from '1' to '0'. Scales from the posterior half of the flank in *Wendichthys* bear pores on the enamel surface, whereas those from the anterior part of the flank lack these pores (Lund & Poplin, 1997: fig. 6). This taxon is scored '1'.

0. absent

1. present

**156.** **[CH 36] Curved ridges along anterior margin of scales:**

Friedman & Blom 2006; Long et al., 2008; Swartz, 2009; Choo, 2011.

0. absent

1. present

**157.** **[CH 76] Scales – anastomosing linear ornament:**

Choo, 2011. This character was proposed by Choo (2011), but no character description was provided. This character is interpreted here are referring to branching and anastomosing ridges on the scale surface, and taxa are recoded accordingly. Ridges often bifurcate on the scales of *Mimipiscis toombsi* (e.g. Gardiner 1984, fig. 140b) and *M. bartrami* (e.g. Choo, 2011, fig. 17a), and as such the coding for these taxa is revised from ‘0’ to ‘1’. Gardiner (1984: p. 387, fig. 141) describes the scale ornament of *Moythomasia durgaringa* as 'ridges of ganoine which branch and anastomose', and the coding is therefore revised from ‘0’ to ‘1’. The coding for *Mansfieldiscus* (Long, 1988) is also changed from ‘0’ to ‘1’. The coding for *Krasnoyarichthys* (Prokofiev, 2002) is revised from ‘?’ to ‘1’. The coding for *Cheirolepis canadensis* (Arratia & Cloutier, 1996), C. trailli (Pearson & Westoll, 1979) and *Donnrosenia* (Long et al., 2008) is revised from ‘1’ to ‘0’.

0. absent

1. present

**158.** **Lepidotrichia:**

Friedman, 2007; Brazeau, 2009; Zhu et al., 2009; Friedman & Brazeau, 2010; Davis et al., 2012; Zhu et al., 2013; Brazeau & Friedman, 2014; Giles et al., 2015b.

0. absent

1. present

**159.** **[CH 37] Fringing fulcra:**

Patterson, 1982; Gardiner & Schaeffer, 1989; Coates, 1999; Dietze, 2000; Schultze & Cumbaa, 2001; Cloutier & Arratia, 2004; Friedman & Blom, 2006; Friedman, 2007; Long et al., 2008; Swartz, 2009; Zhu et al., 2009; Choo, 2011; Xu & Gao, 2011; Zhu et al., 2013; Xu et al., 2014.

0. absent

1. present

**160.** **Dorsal margin of cleithrum:**

Cloutier & Ahlberg, 1996; Schultze & Cumbaa, 2001; Zhu & Schultze, 2001; Zhu et al., 2001; Zhu & Yu, 2002; Cloutier & Arratia, 2004; Zhu et al., 2006; Friedman, 2007; Zhu et al., 2009; Giles et al., 2015b.

0. pointed

1. broad and rounded

**161.** **Anocleithrum:**

Gardiner & Schaeffer, 1989; Lund et al., 1995; Cloutier & Ahlberg, 1996; Dietze, 2000; Poplin & Lund, 2000; Schultze & Cumbaa, 2001; Zhu & Schultze, 2001; Zhu et al., 2001; Zhu & Yu, 2002; Cloutier & Arratia, 2004; Zhu et al., 2006; Friedman, 2007; Zhu et al., 2009; Zhu et al., 2013.

0. bone developed as postcleithrum

1. bone developed as anocleithrum sensu stricto

2. bone absent

**162.** **[CH 39] Presupracleithrum:**

Patterson, 1982; Gardiner, 1984; Gardiner & Schaeffer, 1989; Taverne, 1997; Lund, 2000; Schultze & Cumbaa, 2001; Zhu & Schultze, 2001; Zhu et al., 2001; Lund & Poplin, 2002; Zhu & Yu, 2002; Cloutier & Arratia, 2004; Gardiner et al., 2005; Friedman & Blom, 2006; Zhu et al., 2006; Friedman, 2007; Long et al., 2008; Swartz, 2009; Zhu et al., 2009; Choo, 2011; Xu & Gao, 2011; Zhu et al., 2013; Xu et al., 2014. Presence of a presupracleithrum is only inferred in *Donnrosenia* (Long et al., 2008), *Gogosardina* (Choo et al., 2009) and *Kentuckia hlavini* (Dunkle, 1964), and the coding for these taxa is revised from ‘1’ to ‘?’. Similarly, absence is inferred in *Krasnoyarichthys* (Prokofiev, 2002) and *Novagonatodus* (Long, 1988; Holland et al., 2007), and the coding is thus changed from '0' to '?'. An elongate bone termed the 'anocleithrum' is variably present in *Wendichthys* (Lund & Poplin, 1997) in the position occupied by the presupracleithrum in other taxa. We regard this as a positional homologue, and code the taxon '0/1' to reflect this polymorphism. Coded as '?' in *C. trailli* following arguments in Friedman & Blom (2006). The coding is revised to '0' in *Osorioichthys* (Taverne, 1997). The coding for *Moythomasia lineata* is revised to '1' (Choo, 2015).

0. absent

1. present

**163.** **Perforate propterygium:**

Patterson, 1982; Gardiner, 1984; Gardiner & Schaeffer, 1989; Rosen, 1989; Taverne, 1997; Coates, 1999; Zhu & Schultze, 2001; Zhu et al., 2001; Zhu & Yu, 2002; Zhu et al., 2006; Brazeau, 2009; Zhu et al., 2009; Friedman & Brazeau, 2010; Xu & Gao, 2011; Davis et al., 2012; Zhu et al., 2013; Xu et al., 2014; Giles et al., 2015b.

0. absent

1. present

**164.** **[CH 41] Anterior rays embrace propterygium:**

Patterson, 1982; Gardiner, 1984; Gardiner & Schaeffer, 1989; Taverne, 1997; Coates, 1999; Schultze & Cumbaa, 2001; Zhu & Schultze, 2001; Friedman & Blom, 2006; Long et al., 2008; Swartz, 2009; Choo, 2011; Xu & Gao, 2011. The radials are only described for *Mimipiscis toombsi*, so the coding for this character is revised from '1’ to ‘?’ for *M. bartrami* (Choo, 2011). The radials are not described in *Gogosardina* (Choo et al., 2009), so the coding is changed from ‘1’ to ‘?’.

0. absent

1. present

**165.** **[CH 42] Metapterygium elongated relative to preceding radials:**

Taverne, 1997; Friedman & Blom, 2006; Long et al., 2008; Swartz, 2009; Xu & Gao, 2011; Xu et al., 2014. The endoskeletal shoulder girdle is only described for *Mimipiscis toombsi*, so the coding for this character is revised to '?' for *M. bartrami* (Gardiner, 1984; Choo, 2011). The metapterygium is not described in *Gogosardina* (Choo et al., 2009), *Howqualepis* (Long, 1988) or *Moythomasia durgaringa* (Gardiner, 1984), so the coding is changed from ‘1’ to ‘?’. Although Swartz (2009) describes a series of endoskeletal radials in *Stegotrachelus*, the elements figured in fig. 17 have a scale-like morphology and appear to be made of dermal bone. As such, the coding in this taxon is revised from ‘1’ to ‘?’. The coding in *Cheirolepis trailli* is revised from ‘0’ to ‘1’ (Giles et al., 2015a).

0. absent

1. present

**166.** **Pectoral fin radials:**

Zhu & Yu, 2002; Friedman, 2007. Two series of pectoral fin radials are described (but not figured) for *Cheirolepis candensis* (Arratia & Cloutier, 2004). Although we consider this arrangement to be unlikely, for now this taxon is coded '1'. Although Swartz (2009) describes a series of endoskeletal radials in *Stegotrachelus*, the elements figured in fig. 17 have a scale-like morphology and appear to be made of dermal bone. As such, this taxon is coded '?'.

0. unjointed

1. jointed

**167.** **[CH 40] Triradiate scapulocoracoid:**

Zhu & Schultze, 2001; Zhu et al., 2001; Zhu & Yu, 2002; Zhu et al., 2006; Friedman, 2007; Zhu et al., 2009; Xu & Gao, 2011; Zhu et al., 2013; Xu et al., 2014. The endoskeletal shoulder girdle is only described for *Mimipiscis* *toombsi*, so the coding for this character is revised from '?' for *M. bartrami* (Gardiner, 1984; Choo, 2011). The precise morphology of the scapulocoracoid is not known for *Cheirolepis canadensis* (Arratia & Cloutier, 1996) or *Gogosardina* (Choo et al., 2009), and as such the coding for these taxa is changed from ‘0’ to ‘?’. *Cheirolepis trailli* is coded ‘0’ (Giles et al., 2015a).

0. absent

1. present

**168.** **[CH 43] Pectoral fin endoskeleton:**

Taverne, 1997; Coates, 1999; Friedman & Blom, 2006; Long et al., 2008; Swartz, 2009; Xu & Gao, 2011; Xu et al., 2014. The pectoral fin of *Cuneognathus* (Friedman & Blom, 2006) and *Kentuckia hlavini* (Dunkle, 1964) is unknown, and so the coding is revised from ‘1’ to ‘?’.

0. extends far beyond body wall (fins lobate)

1. barely extends beyond body wall (fins not lobate)

**169.** **Fin articulation:**

Zhu & Schultze, 2001; Zhu et al., 2001; Zhu & Yu, 2002; Zhu et al., 2006; Friedman, 2007; Zhu et al., 2009; Friedman & Brazeau, 2010; Zhu et al., 2013; Giles et al., 2015b.

0. monobasal

1. polybasal

**170.** **Pectoral fin morphology:**

0. leaf-like

1. not leaf-like

**171.** **[CH 44] Pectoral fin-ray segmentation*****:**

Coates, 1999; Friedman & Blom, 2006; Long et al., 2008; Choo, 2011; Xu & Gao, 2011; Xu et al., 2014. The pectoral fin in *Kentuckia hlavini* (Dunkle, 1964) is not preserved, so the coding for this taxon is revised from ‘1’ to ‘?’. The coding for *Osorioichthys* (Taverne, 1997) is revised from '0' to '1'. The segmentation of the pectoral fin is not described for *Limnomis* (Daeschler, 2000), so the coding for this taxon is revised from '0' to '?'.

0. roughly even segmentation to fin base

1. proximal segments elongate with terminal segmentation

2. no significant segmentation on pectoral fin

**172.** **Paired fin spines:**

Zhu et al., 2001; Zhu & Yu, 2002; Friedman, 2007; Zhu et al., 2009; Brazeau, 2009; Davis et al., 2012; Zhu et al., 2013; Giles et al., 2015b.

0. absent

1. present

**173.** **[CH 38] Pelvic fins:**

Friedman & Blom, 2006; Friedman, 2007; Brazeau, 2009; Choo, 2011; Davis et al., 2012; Zhu et al., 2013; Brazeau & Friedman, 2014; Giles et al., 2015b.

0. absent

1. present

**174.** **[CH 45] Pelvic fin insertion:**

Gardiner & Schaeffer, 1989; Coates, 1998; Coates, 1999; Lund, 2000; Schultze & Cumbaa, 2001; Cloutier & Arratia, 2004; Friedman & Blom, 2006; Zhu et al., 2006; Long et al., 2008; Swartz, 2009; Zhu et al., 2009; Choo, 2011; Xu et al., 2014. The pelvic fin is incomplete in *Novagonatodus* (Long, 1988; Holland et al., 2007), so this taxon is coded '?'. The coding for *Moythomasia lineata* is revised to '0' (Choo, 2015).

0. shorter than fin depth (short based)

1. longer than fin depth (long based)

**175.** **[CH 46] Epichordal lobe of caudal fin:**

Patterson, 1982; Cloutier & Ahlberg, 1996; Coates, 1999; Schultze & Cumbaa, 2001; Zhu & Schultze, 2001; Friedman & Blom, 2006; Long et al., 2008; Swartz, 2009; Choo, 2011.

0. present

1. absent

**176.** **[CH 47] Fulcra along dorsal ridge of caudal fin:**

Patterson, 1982; Taverne, 1997; Gardiner & Schaeffer, 1989; Gardiner et al., 2005; Friedman & Blom, 2006; Long et al., 2008; Choo, 2011. Choo's (2011) codes for this character appear reversed.

0. absent

1. present

**177.** **Basal scutes on fins:**

Zhu & Yu, 2002; Friedman, 2007.

0. absent

1. present

**178.** **[CH 48] Dorsal scutes anterior to dorsal fin*****:**

Lund, 2000; Poplin & Lund, 2000; Cloutier & Arratia, 2004; Friedman & Blom, 2006; Long et al., 2008; Swartz, 2009; Choo; 2011. Choo's (2011) codes for this character appear reversed.

0. absent

1. few limited to region immediately anterior to fin (basal fulcra only)

2. many, extending to posterior of skull roof (complete set of dorsal ridge scales)

**179.** **[CH 49] Ventral scutes between hypochordal lobe of caudal fin and anal fin:**

Patterson, 1982; Taverne, 1997; Friedman & Blom, 2006; Long et al., 2008; Choo, 2011. The coding for *Howqualepis rostridens* (Long, 1988) and *Melanecta* (Coates, 1998) is revised from ‘0’ to ‘1’.

0. absent

1. present

**180.** **[CH 50] Ventral scutes anterior to anal fin:**

Cloutier & Arratia, 2004; Friedman & Blom, 2006; Long et al., 2008; Swartz, 2009; Choo, 2011. The coding for *Gogosardina* (Choo et al., 2009) is revised from ‘1’ to ‘0’. *Cuneognathus* (Friedman & Blom, 2006) possesses several scutes immediately anterior to the anal fin, although it is unclear how far anteriorly they extend. As such, the coding is changed from ‘?’ to ‘1’. The coding in *Melanecta* (Coates, 1998) is revised from ‘?’ to ‘1’. Choo (2011) erroneously codes *Krasnoyarichthys* (Prokofiev, 2002) as state ‘2’, which lacks a description; the coding is revised to ‘1’. The coding for *Moythomasia lineata* is revised to '0' (Choo, 2015).

0. absent

1. present

**181.** **[CH 52] Dorsal fin(s):**

Gardiner & Schaeffer, 1989; Schultze & Cumbaa, 2001; Zhu & Schultze, 2001; Zhu et al., 2001; Zhu & Yu, 2002; Cloutier & Arratia, 2004; Friedman & Blom, 2006; Zhu et al., 2006; Friedman, 2007; Long et al., 2008; Brazeau, 2009; Swartz, 2009; Zhu et al., 2009; Choo, 2011; Davis et al., 2012; Zhu et al., 2013; Giles et al., 2015b.

0. two

1. one

**182.** **[CH 51] Relative positions of anal and (second) dorsal fin*****:**

Poplin & Lund, 2000; Cloutier & Arratia, 2004; Friedman & Blom, 2006; Long et al., 2008; Swartz, 2009; Choo, 2011. The coding for *Stegotrachelus* is revised from ‘1’ to ‘2’.

0. anal shifted anteriorly relative to dorsal

1. fins opposite one another

2. anal shifted posteriorly relative to dorsal

**182.** **Preoperculum - vertical linear ornament:**

Choo, 2011. The coding is changed to ‘0’ for taxa where the preoperculum is not preserved or the ornament is not preserved sufficiently to discern orientation. Vertical ornament is present on the preopercular of *Cheirolepis* *trailli* (Pearson & Westoll, Fig 7b, Plate IIA), *C. canadensis* (Arratia 1996 fig. 11) and *Kansasiella* (Poplin 1974 Plate II), and the codings for these taxa are changed to ‘1’.

0. absent

1. present

**B. Deleted Characters**

**[CH 2] Premaxillae - shape of (0) wider than deep; (1) depth approximately equal to width.**

This character is deleted as the premaxillae are often poorly preserved or distorted in early osteichthyans, with the result that the shape is highly vulnerable to taphonomic distortions.

**[CH 31]** **Scale rows, number of (0) fewer than 60; (1) more than 60.**

This character is deleted due to a difficulty in counting the number of scale rows in many taxa, and because the distinction between the two states relies on an arbitrary number.

**[CH 55] Intertemporal - contact with parietal (0) no contact; (1) contacts parietal laterally; (2) contacts parietal posterolaterally.**

This character is deleted due to the subjective judgement required when considering whether the contact is lateral or posterolateral.

**[CH 56] Rostral - shape of (0) widens anteriorly; (1) equal anteriorly and posteriorly; (2) narrows anteriorly.**

This character is deleted because the anteriormost portion of the snout, comprising the rostral, is often poorly preserved in actinopts, and distortion or poor preservation may cause the shape of the rostral to be midleading.

**[CH 66] Serrated linear ganoine ornament on skull roof (0) absent; (1) present.**

This character was erected by Choo (2011) to account for the ‘herringbone’ ornament on the skull roof of *Moythomasia*. This character is difficult to assess. Furthermore, ‘herringbone’ ornament is identified only on the skull roof of *Moy. durgaringa* (Gardiner, 1984), and does not appear to be present on the skull roof of *Moy. nitida* or *Moy. perforata* (Jessen, 1968).

**[CH 77] Maxilla - shape of anterior section (0) straight; (1) reflexed.**

Although listed as a new character by Choo (2011), no description is given and this character is not coded in Choo’s matrix. Thus the rationale behind the character is unclear.

**2) List of Taxa**

| **Taxon** | **Source** | **Specimens** |
| --- | --- | --- |
| *Acanthodes bronni* | Miles, 1973; Davis et al., 2012. |  |
| *Boreosomus piveteau* | Nielsen, 1942. |  |
| *Cheirolepis canadensis* | Arratia & Cloutier, 1996; Arratia, 2009. |  |
| *Cheirolepis schultzei* | Arratia & Cloutier, 2004. |  |
| *Cheirolepis trailli* | Pearson & Westoll, 1979; Giles et al., 2015a. |  |
| *Cladodoides wildungensis* | Maisey, 2005. |  |
| *Coccocephalichthys wildi* | Poplin, 1974; Poplin & Véran, 1996. |  |
| *Cosmoptychius wildi* | Schaeffer, 1971; Coates, 1999 |  |
| *Cuneognathus gardineri* | Friedman & Blom, 2006. |  |
| *Dialipina salgueiroensis* | Schultze, 1968; Schultze & Cumbaa, 2001. |  |
| *Diplocercides kayseri* | Stensiö, 1922a,b; Forey, 1996. |  |
| *Donnrosenia schaefferi* | Long et al., 2008. |  |
| *Entelognathus primordialis* | Zhu et al., 2013. |  |
| *Eusthenopteron foordi* | Jarvik, 1980. |  |
| *Gogonasus andrewsae* | Long et al., 1997; Long et al., 2006; Holland, 2014. |  |
| *Gogosardina coatesi* | Choo et al., 2009. |  |
| *Glyptolepis groenlandica* | Jarvik, 1972; Jarvik, 1980. |  |
| *Guiyu oneiros* | Zhu et al., 2009; Qiao & Zhu, 2010. |  |
| *Howqualepis rostridens* | Long, 1988; Choo, 2009. |  |
| *Kansasiella eatoni* | Poplin, 1974 |  |
| *Kentuckia deani* | Rayner, 1951; Giles & Friedman, 2014. | MCZ 8363, MCZ 5226 |
| *Kentuckia hlavini* | Dunkle, 1964; Feldman, 1996. |  |
| *Krasnoyarichthys jesseni* | Poplin, 1974. |  |
| *Lawrenciella schaefferi* | Poplin, 1984; Hamel & Poplin, 2008. |  |
| *“Ligulalepis”* | Basden et al., 2000; Basden & Young, 2001. |  |
| *Limnomis delayni* | Daeschler, 2000. |  |
| *Luederia kempi* | Schaeffer & Dalquest, 1978. |  |
| *Mansfieldiscus sweeti* | Long, 1988. |  |
| *Meemannia eos* | Zhu et al., 2006, 2010. |  |
| *Melanecta anneae* | Coates, 1998. |  |
| *Mesopoma planti* | Coates, 1999. |  |
| *Miguashaia bureaui* | Cloutier, 1996. |  |
| *Mimipiscis bartrami* | Gardiner, 1984: Choo, 2011. |  |
| *Mimipiscis toombsi* | Gardiner, 1984; Choo, 2011; Giles & Friedman, 2014. |  |
| *Moythomasia durgaringa* | Gardiner, 1984; Long & Trinajstic, 2010; Choo, 2015. |  |
| *Moythomasia nitida* | Jessen, 1968. |  |
| *Moythomasia lineata* | Jessen, 1968; Choo, 2015. |  |
| *Novagonatodus kasantsevae* | Long, 1998; Holland et al., 2007. |  |
| *Osorioichthys marginis* | Taverne, 1997. | IRSNB P.1340 |
| *Onychodus jandemarrai* | Andrews et al., 2006. |  |
| *Osteolepis macrolepidotus* | Jarvik 1948 |  |
| *Porolepis sp.* | Jarvik, 1972; Jarvik, 1980. |  |
| *Psarolepis romeri* | Yu, 1998; Zhu et al., 1999; Zhu & Yu, 2009; Qu et al., 2013. |  |
| *Pteronisculus stensioi* | Nielsen 1942; Coates, 1998. |  |
| *Stegotrachelus finlayi* | Gardiner, 1963; Swartz, 2009. |  |
| *Styloichthys changae* | Zhu & Yu, 2002; Zhu et al., 2006; Friedman, 2007. |  |
| *Tegeolepis clarki* | Gardiner, 1963; Dunkle & Schaeffer, 1973. | TOOTH SPECIMEN |
| *Wendichthys dicksoni* | Lund & Poplin, 1997. |  |
| *Woodichthys bearsdeni* | Coates, 1998. |  |

**Part C. Supplementary References**

Ahlberg, P. E. and J. A. Clack. 1998. Lower jaws, lower tetrapods - a review based on the Devonian genus Acanthostega. *Transactions of the Royal Society of Edinburgh: Earth Sciences* **88**: 11–46.

Ahlberg, P. E. and Johanson, Z. 1998. Osteolepiforms and the ancestry of tetrapods. *Nature* **395**: 792–794.

Ahlberg, P. E., Lukševičs, E. and Mark-Kurik, E. 2000. A near-tetrapod from the Baltic Middle Devonian. *Palaeontology* **43**: 533–548.

ANDREWS, S. M., LONG JA, AHLBERG P.E., BARWICK R. and CAMPBELL, K. 2006. The structure of the sarcopterygian *Onychodus jandemarrai* n. sp. from Gogo, Western Australia: with a functional interpretation of the skeleton. *Transactions of the Royal Society of Edinburgh* **96**: 197–307.

ARRATIA, G. 2009. Identifying patterns of diversity of the actinopterygian fulcra. *Acta Zoologica*, *Supplement* **90**: 220–235.

ARRATIA, G. and CLOUTIER, R. 1996. Reassessment of the morphology of *Cheirolepis canadensis* (Actinopterygii). 165–197. *In* SCHULTZE, H.-P. and CLOUTIER, R. (eds). *Devonian fishes and plants of Miguasha, Quebec, Canada*. Verlag Dr. Frederich Pfeil, Munich, 374 pp.

BASDEN, A. M. and YOUNG, G. C. 2001. A primitive actinopterygian neurocranium from the Early Devonian of southeastern Australia. *Journal of Vertebrate Paleontology*, **21**: 754–766.

BASDEN, A. M., YOUNG, G. C., COATES, M. I. and RITCHIE, A. 2000. The most primitive osteichthyan braincase? *Nature*, **403**: 185–188.

Brazeau, M. D. 2009. The braincase and jaws of a Devonian ‘acanthodian’ and modern gnathostome origins. *Nature* **457**: 305–308.

BRICE, D. 1988. *Le Dévonien de Ferques. Bas-Boulonnais (N.France). Paléontologie - Sédimentologie - Stratigraphie - Tectonique.* Coll. Biostratigraphie du Paléozoïque, Université de Bretagne Occidentale, Brest, 522 pp.

CHOO, B. 2011. Revision of the actinopterygian genus *Mimipiscis* (=*Mimia*) from the Upper Devonian Gogo Formation of Western Australia and the interrelationships of the early Actinopterygii. *Earth and Environmental Science Transactions of the Royal Society of Edinburgh* **102**: 77–104.

CHOO, B., LONG, J. A. and TRINAJSTIC, K. 2009. A new genus and species of basal actinopterygian fish from the Upper Devonian Gogo Formation of Western Australia. *Acta Zoologica*, *Supplement* **90**: 194–210.

Cloutier, R. and Ahlberg, P. E. 1996. Morphology, characters, and the interrelationships of basal sarcopterygians. 445–479. *In* (Stiassny, M. L. J., Parenti, L. R. and Johnson, G. D. (eds) *Interrelationships of Fishes.* Academic Press, 496 pp.

Cloutier, R. 1996. The primitive actinistian *Miguashaia bureaui* Schultze (Sarcopterygii). 227–247. *In* SCHULTZE, H.P., and CLOUTIER, R. (eds) *Devonian Fishes and Plants of Miguasha, Quebec, Canada.* Verlag Dr. Friedrich Pfeil, Munich, 374 pp.

CLOUTIER, R. and ARRATIA, G. 2004. Early diversification of actinopterygians. 217–270. *In* ARRATIA, G., WILSON, M. V. H. and CLOUTIER, R. (eds). *Recent advances in the origin and early radiation of vertebrates*. Verlag Dr. Friedrich Pfeil, Munich, 703 pp.

COATES, M. I. 1998. Actinopterygians from the Namurian of Bearsden, Scotland, with comments on early actinopterygian neurocrania. *Zoological Journal of the Linnean Society* **122**: 27–59.

COATES, M. I. 1999. Endocranial preservation of a Carboniferous actinopterygian from Lancashire, UK, and the interrelationships of primitive actinopterygians. *Philosophical Transactions of the Royal Society of London B* **354**: 435–462.

COATES, M. I. and SEQUEIRA, S. E. K. 1998. The braincase of a primitive shark. *Transactions of the Royal Society of Edinburgh: Earth Sciences* **89**: 63–85.

COATES, M. I. and SEQUEIRA, S. E. K. 2001a. A new stethacanthid chondrichthyans from the Lower Carbonifeous of Bearsden, Scotland. *Journal of Vertebrate Paleontology* **21**: 438–459.

DAESCHLER, E. B. 2000. An early actinopterygian fish from the Catskill Formation (Late Devonian, Famennian) in Pennsylvania, U.S.A. *Proceedings of the Academy of Natural Sciences of Philadelphia* **150**: 181–192.

Daeschler, E. B., Shubin, N. H. and Jenkins, F. A. 2006. A Devonian tetrapod-like fish and the evolution of the tetrapod body plan. *Nature* **440**: 757–763.

Darras, L. 2006. Vertébrés du Dévonien du Boulonnais: données nouvelles pour les formations de Blacourt et de Ferques. Unpublished Master’s thesis, University of Lille.

Davis, S. P., Finarelli, J. A. and Coates, M. I. 2012. *Acanthodes* and shark-like conditions in the last common ancestor of modern gnathostomes. *Nature* **486**: 247–250.

DERYCKE, C., BRICE, D., BLIECK, A. and MOURAVIEFF, N. 1995. Upper Givetian and Frasnian ichthyloliths from Bas-Boulonnais (Pas-de-Calais, France): preliminary records. *Bulletin du Muséum national d'histoire naturelle. 4e Série. Section C. Sciences de la Terre. Paléontologie, Géologie, Minéralogie* **17**: 487–511.

Derycke-Khatir C. 2005. Microrestes de Vertébrés du Paléozoïque supérieur de la Manche au Rhin. *Société Géologique du Nord* **33**: 1–261.

Dietze, K. 2000. A revision of paramblypterid and amblypterid actinopterygians from Upper Carboniferous–Lower Permian lacustrine deposits of Central Europe. *Palaeontology* **43**: 927–966.

Dunkle, D. H. 1964. Preliminary description of a paleoniscoid fish from the Upper Devonian of Ohio. *Cleveland Museum of Natural History* **3**: 1–16.

Feldman, R. M. (ed.) 1996 - *Fossils of Ohio*. State of Ohio Division of Geological Survey, Ohio. 577 pp.

FRIEDMAN, M. 2007. *Styloichthys* as the oldest coelacanth: implications for early osteichthyan interrelationships. *Journal of Systematic Palaeontology* **5**: 289–343.

FRIEDMAN, M. and BLOM, H. 2006. A new actinopterygian from the Famennian of East Greenland and the interrelationships of Devonian ray-finned fishes. *Journal of Paleontology*, **80**: 1186–1204.

Friedman, M. and Brazeau, M. D. 2010. A reappraisal of the origin and basal radiation of the Osteichthyes. *Journal of Vertebrate Paleontology* **30**: 36–56.

Forey, P. L. 1980. *Latimeria*: a paradoxical fish. *Proceedings of the Royal Society of London B: Biological Sciences* **208**: 369–384.

FOREY, P. L. 1998. *History of the coelacanth fishes.* Chapman & Hall, London, 419 pp.

GARDINER, B.G. 1963. Certain palaeoniscoid fishes and the evolution of the snout in actinopterygians. *Bulletin of the British Museum (Natural History): Geology* **8**: 254–325.

GARDINER, B.G. 1984. The relationships of the palaeoniscid fishes, a review based on new specimens of *Mimia* and *Moythomasia* from the Upper Devonian of Western Australia. *Bulletin of the British Museum (Natural History): Geology* **37**: 173–428.

GARDINER, B.G. and SCHAEFFER, B. 1989. Interrelationships of lower actinopterygian fishes. *Zoological Journal of the Linnaean Society* **97**: 135–187.

GILES, S. and FRIEDMAN, M. 2014. Virtual reconstruction of endocast anatomy in early ray-finned fishes (Osteichthyes, Actinopterygii). *Journal of Paleontology* **88**: 636–651.

GILES, S., COATES, M. I., GARWOOD, R. J., BRAZEAU, M. D., ATWOOD, R., JOHANSON, Z. and FRIEDMAN, M. 2015a. Endoskeletal structure in *Cheirolepis* (Osteichthyes, Actinopterygii), an early ray-finned fish. *Palaeontology*. Published online 15/7/2015.

GILES, S., FRIEDMAN, M. and BRAZEAU, M. D. 2015b. Osteichthyan-like cranial conditions in an Early Devonian stem gnathostome. *Nature*, **520,** 82–85.

Gradstein, F. M., Ogg, J. G. and Schmitz, M. (eds.). 2012. *The Geologic Time Scale 2012, 2-volume set*. Elsevier, 1176 pp.

HAMEL, M.-H. and POPLIN, C. 2008. The braincase anatomy of *Lawrenciella schaefferi*, actinopterygian from the Upper Carboniferous of Kansas (USA). *Journal of Vertebrate Paleontology* **28**: 989–1006.

Holland, T. M., Long, J. A., Warren, A. & GArvey, J. A. 2007. Second specimen of the lower actinopterygian *Novagonatodus kasantsevae* Long 1988 from the Early Carbonieferous of Mansfield, Victoria **118**: 1–10.

HOLLAND, T. 2014. The endocranial anatomy of *Gogonasus andrewsae* Long, 1985 revealed through micro CT-scanning. *Earth and Environmental Science Transactions of the Royal Society of Edinburgh* **105**: 9–34.

Jarvik, E. 1948. On the morphology and taxonomy of the Middle Devonian osteolepid fishes of Scotland. *Kungliga Svenska Vetenskapsakademiens Handligar* **25**: 1–30.

Jarvik, E. 1972. Middle and Upper Devonian Porolepiformes from East Greenland with special reference to *Glyptolepis groenlandica* n.sp. *Meddelelser om Grønland* **187**: 1–295.

JARVIK E. 1980. *Basic structure and evolution of vertebrates. Volume 1*. Academic Press, London, 575 pp.

Jessen, H. 1968. *Moythomasia nitida* Gross und *M*. cf. *striata* Gross, Devonische palaeonisciden aus dem oberen Plattenkalk der Bergish-Gladbach-Paffrather Mulde (Rheinisches Schiefergebirge). *Palaeontographica Abteilung A* **128**: 87–114.

Lauder, G. V. and Liem, K. F. 1983. The evolution and interrelationships of the actinopterygian fishes. *Bulletin of the Museum of Comparative Zoology, Harvard University* **150**: 95–197.

LONG, J. A. 1988. New palaeoniscoid fishes from the Late Devonian and early Carboniferous of Victoria. *Memoir of the Australasian Association of Palaeontologists* **7**: 1–64.

Long, J. A. 1997. Ptyctodontid fishes (Vertebrata, Placodermi) from the Late Devonian Gogo Formation, Western Australia, with a revision of the European genus *Ctenurella* Ørvig, 1960. *Geodiversitas* **19**: 515–555.

Long, J. A., Young, G. C., Holland, T., Senden, T. J. and Fitzgerald, E. M. G. 2006. An exceptional Devonian fish from Australia sheds light on tetrapod origins. *Nature* **444**: 199–202.

LONG, J. A., CHOO, B. and YOUNG, G. C. 2008. A new basal actinopterygian from the Middle Devonian Aztec Siltstone of Antarctica. *Antarctic Science* **20**: 393–412.

LONG, J. A., TRINAJSTIC, K. 2010. The Late Devonian Gogo Formation Lägerstatte of Western Australia: exceptional early vertebrate preservation and diversity. *Annual Review of Earth and Planetary Sciences* **38**: 255–79.

Lund, R. 2000. The new actinopterygian order Guildayichthyiformes from the Lower Carboniferous of Montana (USA). *Geodiversitas* **22**: 171–206.

Lund, R. and Poplin, C. 2002. Cladistic analysis of the relationships of the tarrasiids (Lower Carboniferous Actinopterygians). *Journal of Vertebrate Paleontology* **22**: 480–486.

Lund, R., Poplin, C. and McCarthy, K. 1995. Preliminary analysis of the interrelationships of some Paleozoic actinopterygii. *Geobios* **28**: 215–220.

Lund, R. and Poplin, C. 1997. The rhadinichthyids (paleoniscoid actinopterygians) from the Bear Gulch Limestone of Montana (USA, Lower Carboniferous). *Journal of Vertebrate Paleontology* **17**: 466–486.

MAISEY, J. G. 1986. Heads and tails: a chordate phylogeny. *Cladistics* **2**: 201–256.

Maisey, J. G. 2005. Braincase of the Upper Devonian shark *Cladodoides wildungensis* (Chondrichthyes, Elasmobranchii), with observations on the braincase in early Chondrichthyans. *Bulletin of the American Museum of Natural History* **288**:1–103.

MILES, R. S. 1973. Relationships of acanthodians. 63–103. *In* GREENWOOD, P. H., MILES, R. S. and PATTERSON, C. (eds). *Interrelationships of fishes*. Academic Press, London, 536 pp.

Murray, A. (2012). Reconstruction of a 381 million year old fish braincase and an analysis of the brain. Unpublished Master’s thesis, University of Leicester.

NIELSEN, E. 1942. Studies on Trassic fishes from East Greenland I. *Glaucolepis* and *Boreosomus*. *Meddelelser om Grønland* **146**: 1–309.

PATTERSON, C. 1982. Morphology and interrelationships of primitive actinopterygian fishes. *American Zoologist* **22**: 241–259.

PEARSON, D.M. and WESTOLL, T. S. 1979. The Devonian actinopterygian *Cheirolepis* Agassiz. *Transactions of the Royal Society of Edinburgh* **70**: 337–399.

POPLIN C. 1974. Étude de quelques Paléoniscidés pennsylvaniens du Kansas. *Cahiers de Paléontologie (Section Vertébrés),* Paris, 151 pp.

Poplin, C. M. 1984. *Lawrenciella schaefferi* n. g., n. sp. (Pisces: Actinopterygii) and the use of endocranial characters in the classification of the palaeonisciformes. *Journal of Vertebrate Paleontology* **4**: 413–421.

Poplin, C. and Lund, R. 2000. Two new deep-bodied palaeoniscoid actinopterygians from Bear Gulch (Montana, USA, Lower Carboniferous). *Journal of Vertebrate Paleontology* **20**: 428–449.

POPLIN C. and VÉRAN, M. 1996. A revision of the actinopterygian fish *Coccocephalus wildi* from the Upper Carboniferous of Lancashire. *Special Papers in Palaeontology* **52**: 7–29.

Prokofiev, A. M. 2002. First finding of an articulated actinopterygian skeleton from the Upper Devonian of Siberia and a reappraisal of the family Moythomasiidae Kazantseva, 1971 (Osteichthyes). *Palaeontological Research* **6**: 321–27.

Qiao, T. and Zhu, M. 2010. Cranial morphology of the Silurian sarcopterygian *Guiyu oneiros* (Gnathostomata: Osteichthyes). *Science China Earth Sciences* **53**: 1836–1848.

QU, Q., ZHU, M. and WANG, W. 2013. Scales and dermal skeletal histology of an early bony fish *Psarolepis romeri* and their bearing on the evolution of rhombic scales and hard tissues. *PLoS ONE,* **8**, e61485.

RAYNER, D. H. 1951. On the cranial structure of an early palaeoniscid, *Kentuckia* gen. nov. *Transactions of the Royal Society of Edinburgh* **62**: 58–83.

Schaeffer, B. 1971. The braincase of the holostean fish *Macrepistius*, with comments on neurocranial ossification in the Actinopterygii. American Museum Novitates **2459**.

SCHAEFFER, B. and DALQUEST, W. W. 1978. A palaeonisciform braincase from the Permian of Texas, with comments on cranial fissures and the posterior myodome. *Novitates*: **2658**: 1–15.

Schultze, H. P. 1968. Palaeoniscoidea-Schuppen aus dem Unterdevon Australiens und Kanadas und aus dem Mitteldevon Spitzbergens Bulletin of the British Museum (Natural History) **16**: 343–376.

Schultze, H.-P and Cumbaa, S. L. 2001. *Dialipina* and the characters of basal osteichthyans. 315-332. *In* Ahlberg, P. E. (ed.) *Major Events in Early Vertebrate Evolution.* Taylor & Francis, London. 418 pp.

Stensiö, E.A. 1922a. Über zwei Coelacanthiden aus dem Oberdevon van Wildungen. *Palaeontologischen Zeitschrift* **4**: 167–210.

Stensiö, E.A. 1922b. Notes on certain crossopterygians. *Proceedings of the Zoological Society of London*: **92**: 1241–1271.

SWARTZ, B. A. 2009. Devonian actinopterygian phylogeny and evolution based on a redescription of *Stegotrachelus finlayi*. *Zoological Journal of the Linnean Society* **56**: 750–784.

TAVERNE, L. 1997. *Osorioichthys marginis*, “Paléonisciforme” du Famennien de Belgique, et la phylogénie de Actinoptérygiens dévoniens (Pisces). *Bulletin de l’Institut Royal des Sciences Naturelles de Belgique* **67**: 57–78.

Watson, D. M. S. 1925. The structure of certain palæoniscids and the relationships of that group with other bony fish. *Proceedings of the Zoological Society of London*. **95**: 815–870.

XU, G.‐H. and GAO, K.Q. 2011. A new scanilepiform from the Lower Triassic of northern Gansu Province, China, and phylogenetic relationships of non‐teleostean Actinopterygii. *Zoological Journal of the Linnean Society* **161**: 595–612.

XU, G.-H., GAO, K.-Q. and FINARELLI, J. A. 2014. A revision of the Middle Triassic scanilepiform fish *Fukangichthys longidorsalis* from Xinjiang, China, with comments on the phylogeny of the Actinopteri. *Journal of Vertebrate Paleontology* **34**: 747–759.

YU, X. 1998. A new porolepiform-like fish, *Psarolepis romeri*, gen. et sp. nov. (Sarcopterygii, Osteichthyes) from the Lower Devonian of Yunnan, China. *Journal of Vertebrate Paleontology* **18**: 261–274.

Zhu, M., Yu, X. and JANVIER, P . 1999. A primitive fossil fish sheds light on the origin of bony fishes. *Nature* **397**: 607–610.

Zhu, M. and Ahlberg, P. E. 2004. The origin of the internal nostril of tetrapods. *Nature* **432**: 94–97.

Zhu, M. & Schultze, H. P. 2001. Interrelationships of basal osteichthyans. 289–314 *In* Ahlberg, P.E. (ed). *Major events in early vertebrate evolution: palaeontology, phylogeny, genetics and development*. Taylor & Francis, London, 418 pp.

Zhu, M., Wang, W. and Yu, X. 2010. *Meemannia eos*, a basal sarcopterygian fish from the Lower Devonian of China–expanded description and significance. 199–214. *In* ELLIOT, D. K., MAISEY, J. G., YU, K. and MIAO, D. (eds). *Morphology, Phylogeny and Paleobiogeography of Fossil Fishes*. Verlag, Dr. Friedrich Pfeil, Munich, 472 pp.

Zhu, M. and Yu, X. 2002. A primitive fish close to the common ancestor of tetrapods and lungfish. *Nature* **418**: 767–770.

Zhu, M., Yu, X. and Ahlberg, P. E. 2001. A primitive sarcopterygian fish with an eyestalk. *Nature* **410**: 81–84.

ZHU, M., YU, X., AHLBERG, P. E., CHOO, B., LU, J., QIAO, T., QU, Q., ZHAO, W., JIA, L., BLOM, H. and ZHU, Y. 2013. A Silurian placoderm with osteichthyan-like marginal jaw bones. *Nature*, **502**:188–193.

Zhu, M., Yu, X., Wang, W., Zhao, W. and Jia, L . 2006. A primitive fish provides key characters bearing on deep osteichthyan phylogeny. *Nature* **441**: 77–80.

ZHU, M., ZHAO, W., JIA, L., LU, J., QIAO, T. and QU, Q. 2009. The oldest articulated osteichthyan reveals mosaic gnathostome characters. *Nature*, **458**: 469–474.

Ziegler, W. and Sandberg C. A. 1990. *The Late Devonian standard conodont zonation.* Courier Forschungsinstitut Senckenberg, 115 pp.

Ziegler, W. and Sandberg C. A. 2000. Utility of Palmatolepids and Icriodontids in recognizing Upper Devonian Series, Stage, and possible Substage boundaries. 335–347. *In* Bultynck, P. (ed.). *Subcommission on Devonian Stratigraphy - Recognition of Devonian series and stage boundaries in geological areas*. Courier Forschungsinstitut Senckenberg.
