# Supplementary material for: An exceptionally preserved Late Devonian actinopterygian provides a new model for primitive cranial anatomy in ray-finned fishes
Source: Proc Biol Sci. 2015 Oct 7;282(1816):20151485. doi: 10.1098/rspb.2015.1485 (PMC4614771; doi:10.1098/rspb.2015.1485)
Supplement: Supplementary Figures [file rspb20151485supp2.pdf]

SUPPLEMENTARY FIGURES

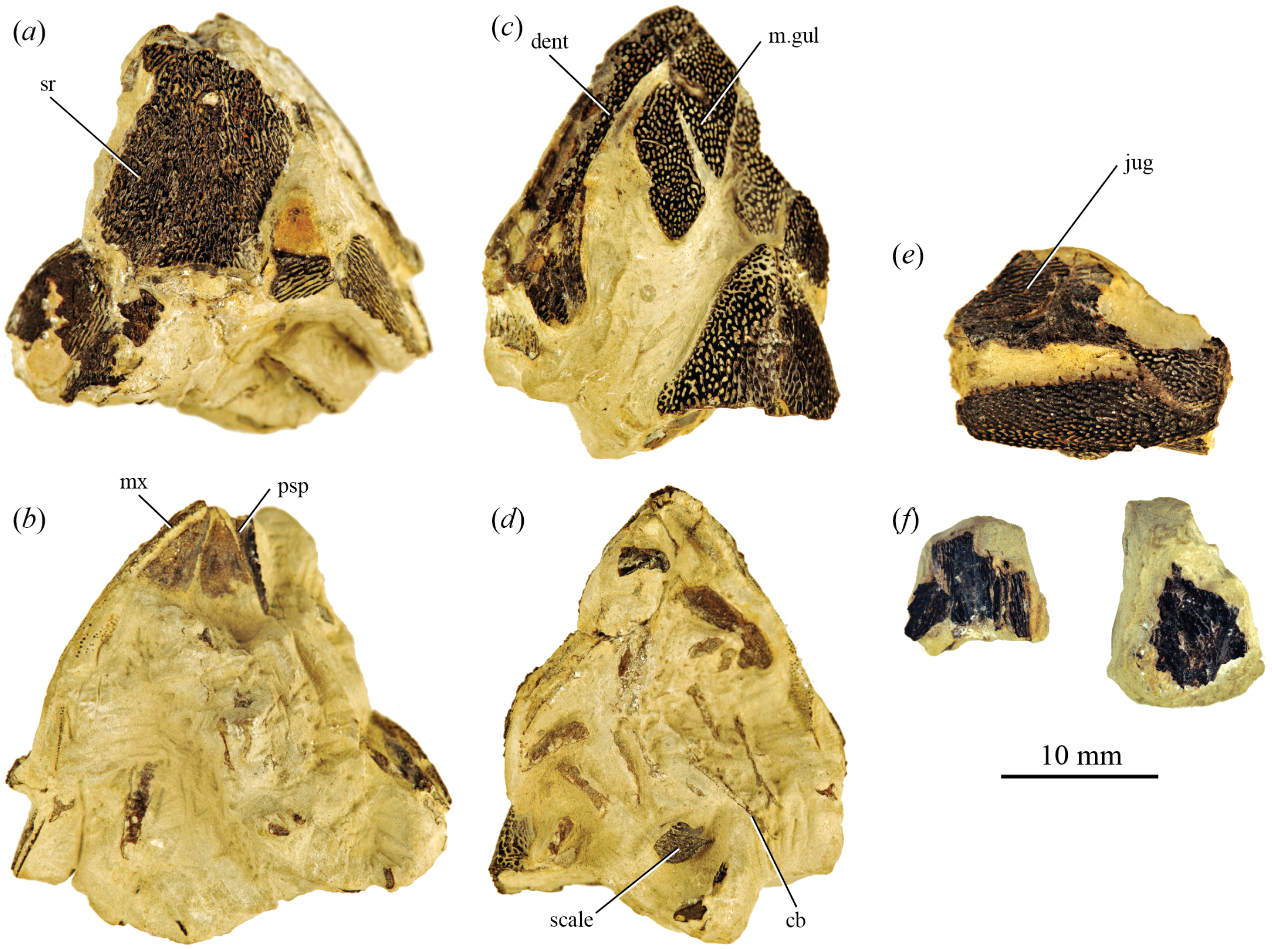

Supplementary Figure 1. Photographs of the fragmented nodule containing the only specimen of *Raynerius splendens* n. gen. et sp. from the Frasnian of France. Largest fragment, containing much of cranium, the braincase and parts of the gill skeleton in dorsal (*a*) and ventral (*b*) view. Fragment containing ventral elements of the skull and parts of the gill skeleton in ventral (*c*) and dorsal (*d*) view. *(e)* Small fragment containing left side of cheek in lateral view. *(f)* Additional fragments containing part of dermal skeleton. Abbreviations cb, ceratobranchial; dent, dentary; jug, jugal; m.gul, median gular; mx, maxilla; psp, parasphenoid; sr, skull roof.

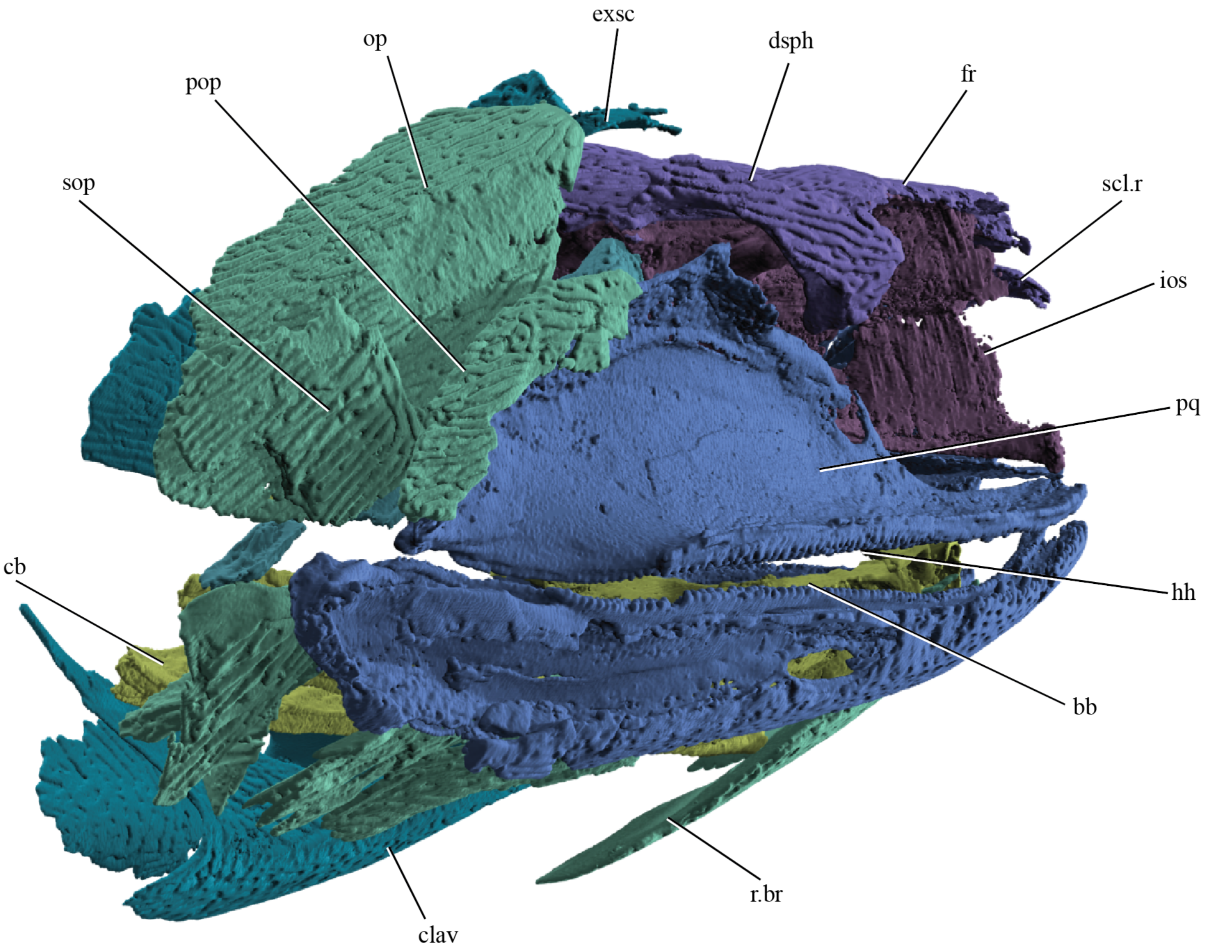

Supplementary Figure 2. *Raynerius splendens* n. gen. et sp. Rendering of specimen in right lateral view (*a*). Abbreviations: bb, basibranchial; cb, ceratobranchial; clav, clavicle; dsph, dermosphenotic; exsc, extrascapular; fr, frontal; hh, hypohyal; ios, interorbital septum; op, operculum; pop, preoperculum; pq, palatoquadrate; r.br, branchiostegal ray scl.r, sclerotic ring; sop, suboperculum. Colour coding of the skeleton: blue, cheek and jaw; purple, skull roof and sclerotic ossicle; pink, braincase; dark green, hyomandibula; light green, operculogular system; turquoise, shoulder girdle; yellow, gill skeleton.

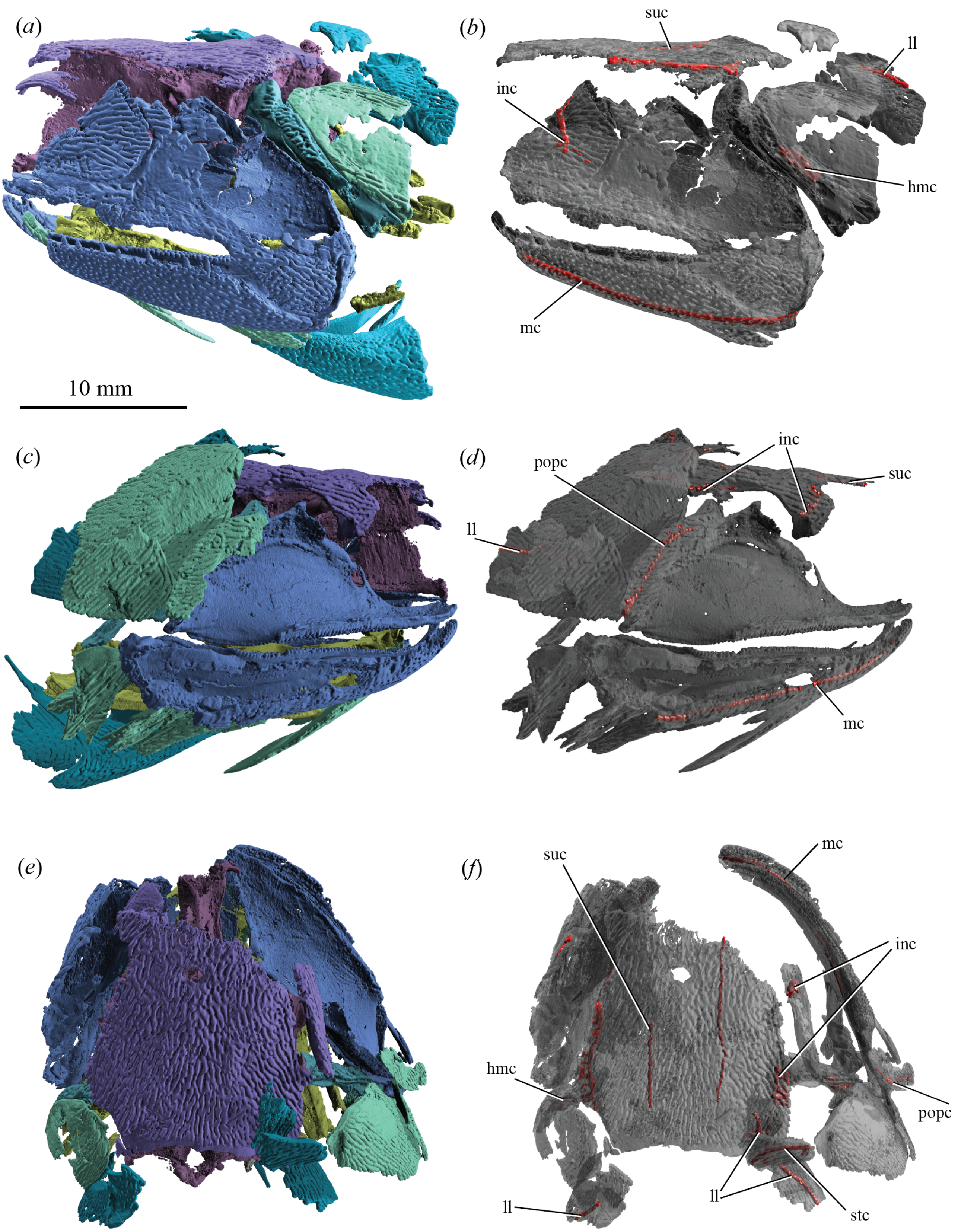

Supplementary Figure 3. Paths of the sensory canals in the dermal bones of *Raynerius splendens* n. gen. et sp. Rendering of specimen in left lateral view (*a*) and in same orientation with dermal bones rendered transparent *(b)*. Rendering *(c)* and transparency *(d)* in right lateral view. Rendering *(e)* and transparency *(f)* in ventral view Abbreviations: inc, infraorbital sensory canal; hmc, hyomandibular canal; ll, lateral line canal; mc, mandibular canal; popc, preopercular sensory canal; stc, supratemporal commissure sensory canal; suc, supraorbital sensory canal.

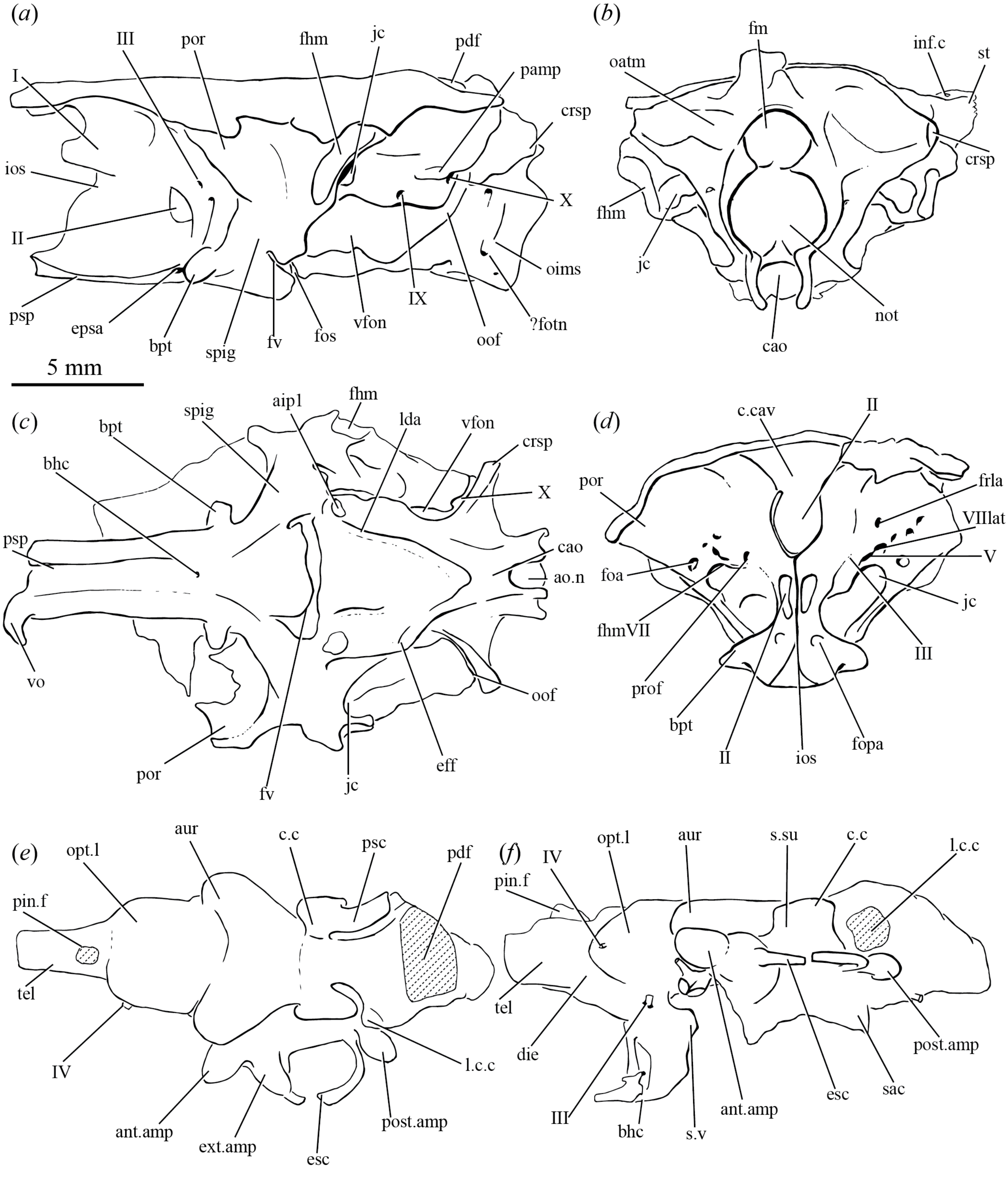

Supplementary Figure 4. Interpretive line drawings of the braincase and endocast of *Raynerius splendens* n. gen. et sp. Braincase in left lateral (a), posterior (b), ventral (c) and anterior (d) view. Partial endocast in dorsal (**e**) and lateral (**f**) view; some details of labyrinth and saccular chamber unresolvable due to poor mineralization. Abbreviations: aip1, articulation of the first infrapharyngobranchial; ant.amp; ampulla of the anterior semicircular canal; ao.n, aortic notch; aur, cerebellar auricle; bhc, buccohypophysial canal; bpt, basipterygoid process; cao, canal for the dorsal aorta; c.cav, cranial cavity; c.c, crus commune; crsp, craniospinal process; die, diencephalon; eff, efferent arteries; epsa, efferent pseudobranchial; esc, external semicircular canal; ext.amp, ampulla of the external semicircular canal; fhm, hyomandibular facet; fhmVII, hyomandibular branch of the facial nerve (continued on next page).

Supplementary Figure 4 continued. fm, foramen magnum; foa, foramen for orbital artery; fopa, ophthalmic artery; fos, otico-sphenoid fossa; ?fotn, foramen for otic nerve; frla, ramus lateralis accessiorus; fv; vental fissure; inf.c, infraorbital canal; ios, interorbital septum; jc, jugular canal; l.c.c, lateral cranial canal; lda, lateral dorsal aorta; not, notochordal tunnel; oatm, origin of anterior trunk muscles; oims, origin of intermuscular septum; oof, oticoccipital fissure; opt. l, optic lobe; pal, palatine nerve; pamp, parampullary process; pdf, posterior dorsal fontanelle; pin, f, pineal foramen; por, postorbital process; post.amp, ampulla of the posterior semicircular canal; prof, profundus nerve; psc, posterior semicircular canal; psp, parasphenoid; sac, sacculus; spig, spiracular groove; s.su, sinus superior; st, supratemporal; s.v, saccus vasculosus; tel, telencephalon; v.fon, vestibular fontanelle; ?vo. vomer; I, olfactory nerve; II, optic nerve; III, oculomotor nerve; IV, trochlear nerve; V, trigeminal nerve; VIIlat, lateralis trunk of facial nerve; fhmVII, hyomandibular branch of the facial nerve; IX, glossopharyngeal nerve; X, vagus nerve.

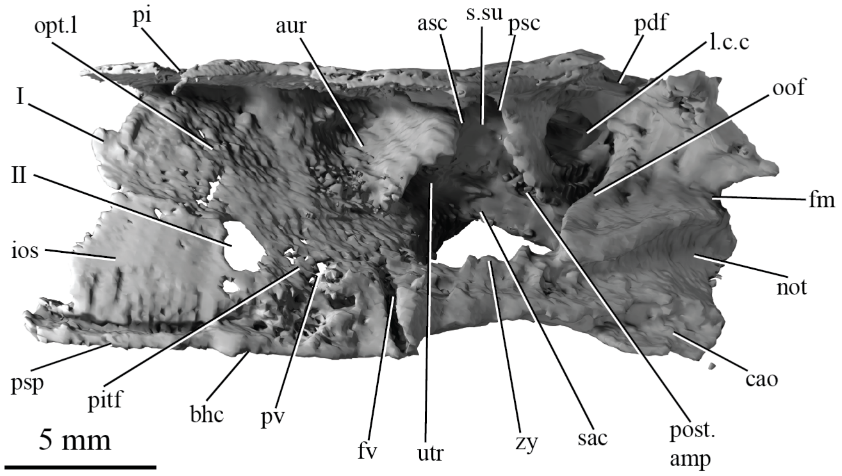

Supplementary Figure 5. Sagittal section through the braincase of *Raynerius splendens* n. gen. et sp. Abbreviations: asc, anterior semicircular canal; aur, cerebellar auricle; bhc, buccohypophysial canal; cao, canal for the dorsal aorta; fm, foramen magnum; fv; vental fissure; ios, interorbital septum; l.c.c, lateral cranial canal; not, notochordal tunnel; oof, oticoccipital fissure; opt.l, optic lobe; pdf, posterior dorsal fontanelle; pi, pineal foramen; pitf, pituitary fossa; post.amp, ampulla of the posterior semicircular canal; psc, posterior semicircular canal; psp, parasphenoid; pv, pituitary vein; sac, sacculus; s.su, sinus superior; utr, utriculus; zy, zygals; I, olfactory nerve; II, optic nerve.

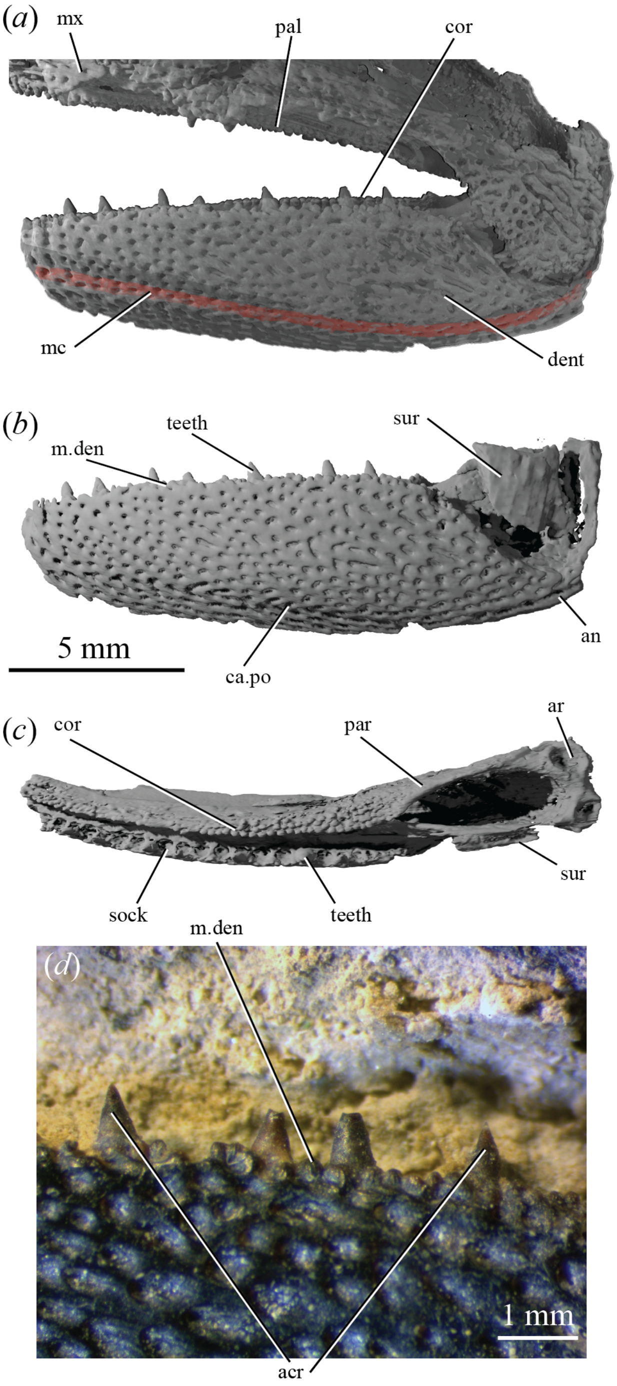

Supplementary Figure 6. Lower jaw and dentition of *Raynerius splendens* n. gen. et sp. *(a)* Rendering of left jaw in left lateral view with dermal bones rendered transparent. *(b)* Lower jaw in lateral view with maxilla removed. *(c)* Lower jaw in dorsal view with maxilla removed. *(d)* Detail of dentition, showing acrodin on the largest laniary teeth. Abbreviations: scr, acrodin; an, angular; ar, articular; ca.po, pores of mandicular canal; cor, coronoids; dent, dentary; mc, mandibular canal; m.den, marginal dentition; mx, maxilla; pal, palate; par, prearticular; sock, empty sockets; sur, surangular; teeth, laniary teeth.

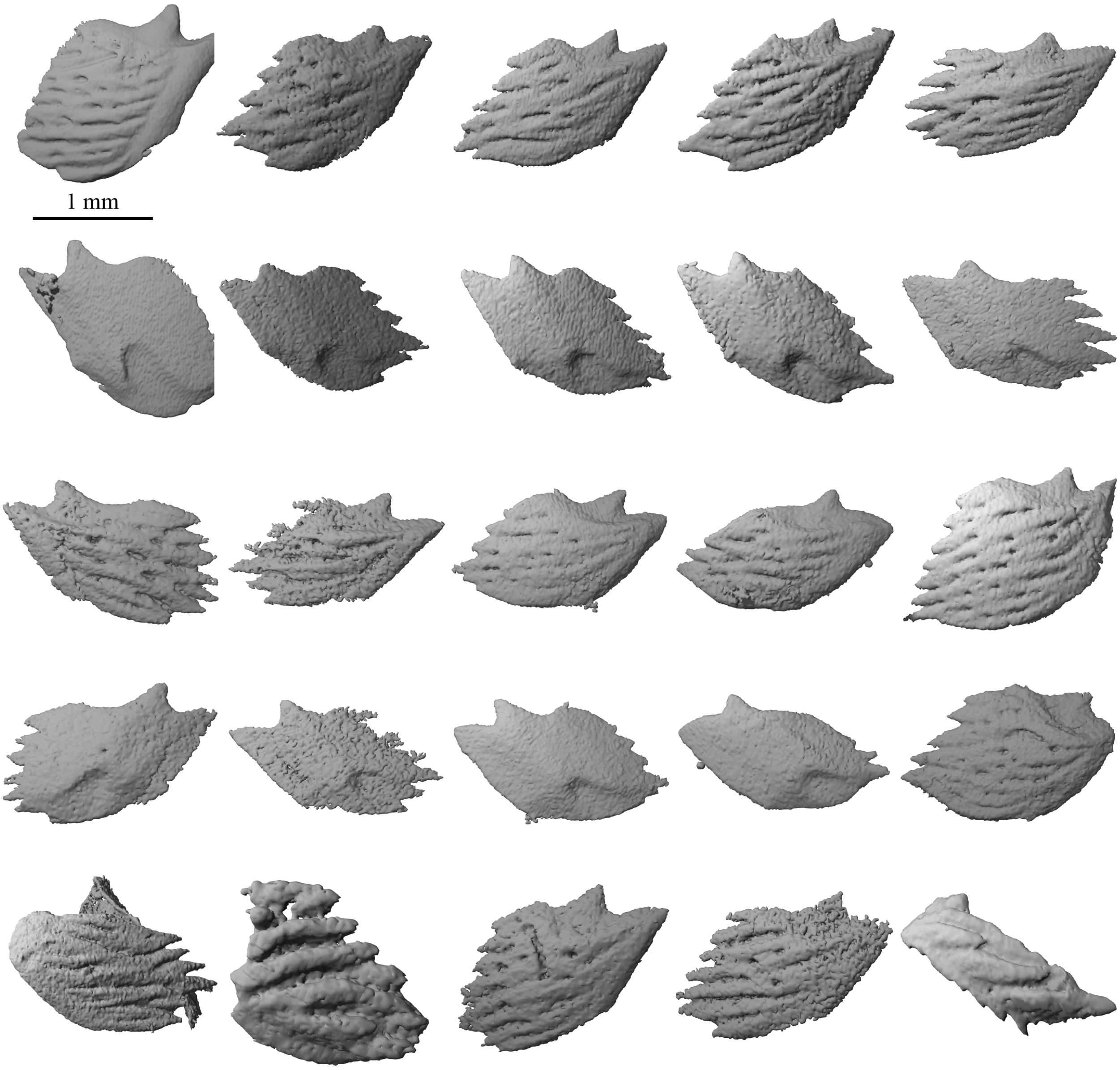

Supplementary Figure 7. Scale morphology of *Raynerius splendens* n. gen. et sp.. Scales are not preserved in articulation, and so their original place on the body is not yet known. All specimens scaled to the same size as scale in the top left of the image (also figured in main text figure 2e and supplementary figure 8); scale bar refers to this scale.

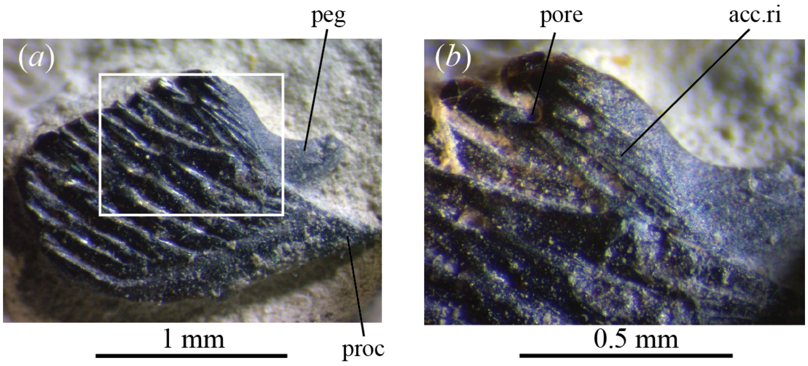

Supplementary Figure 8. Detail of scales of *Raynerius splendens* n. gen. et sp. *(a)* Same scale as shown in main text figure 2e in external view. *(b)* closeup of scale surface (area indicated by white box in panel *a*). Abbreviations: acc.ri, accessory ridges on ornament; peg, peg of scale; pore, pore on scale surface; proc, anterodorsal process.

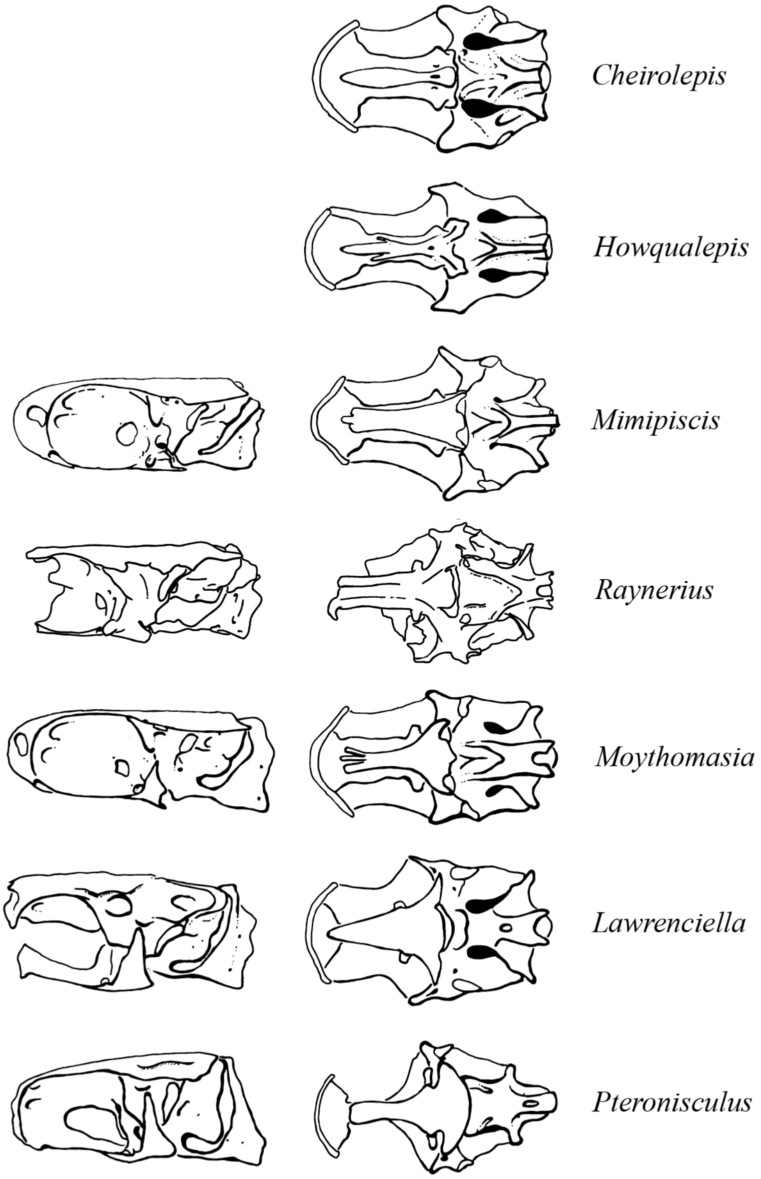

Supplementary Figure 9. Comparative braincase anatomy across early actinopterygians. Braincase images redrawn from: *Cheirolepis*, Giles et al 2015a; *Howqualepis,* Long 1988; *Mimipiscis*, Gardiner 1984, Choo 2011; *Moythomasia*, Long and Trinajstic 2010; *Lawrenciella*, Hamel and Poplin 2008; *Pteronisculus*, Nielsen 1942.

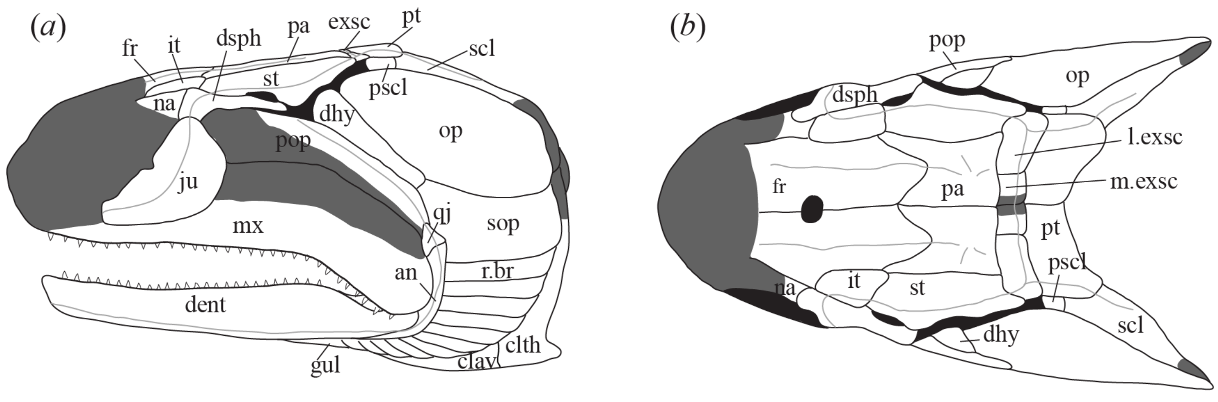

Supplementary Figure 10. Reconstruction of the head of *Raynerius splendens* n. gen. et sp. in lateral *(a)* and dorsal *(b)* views.

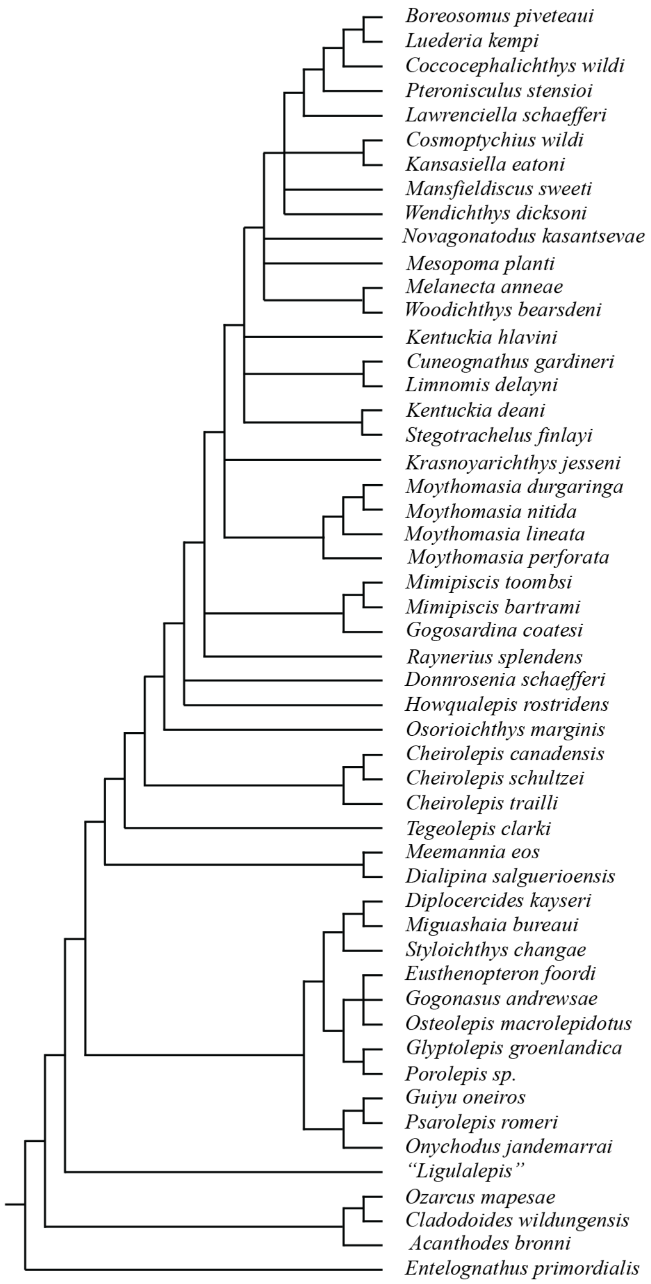

Supplementary Figure 11. Phylogenetic placement of *Raynerius splendens* n. gen. et sp. Adams consensus of 384 most parsimonious trees.
